# Contribution of allelic imbalance to colorectal cancer

**DOI:** 10.1038/s41467-018-06132-1

**Published:** 2018-09-10

**Authors:** Kimmo Palin, Esa Pitkänen, Mikko Turunen, Biswajyoti Sahu, Päivi Pihlajamaa, Teemu Kivioja, Eevi Kaasinen, Niko Välimäki, Ulrika A. Hänninen, Tatiana Cajuso, Mervi Aavikko, Sari Tuupanen, Outi Kilpivaara, Linda van den Berg, Johanna Kondelin, Tomas Tanskanen, Riku Katainen, Marta Grau, Heli Rauanheimo, Roosa-Maria Plaketti, Aurora Taira, Päivi Sulo, Tuomo Hartonen, Kashyap Dave, Bernhard Schmierer, Sandeep Botla, Maria Sokolova, Anna Vähärautio, Kornelia Gladysz, Halit Ongen, Emmanouil Dermitzakis, Jesper Bertram Bramsen, Torben Falck Ørntoft, Claus Lindbjerg Andersen, Ari Ristimäki, Anna Lepistö, Laura Renkonen-Sinisalo, Jukka-Pekka Mecklin, Jussi Taipale, Lauri A. Aaltonen

**Affiliations:** 1 Department of Medical and Clinical Genetics, Medicum, University of Helsinki, Biomedicum Helsinki, PO Box 63 (Haartmaninkatu 8), FI-00014 Helsinki, Finland; 2 Genome-Scale Biology Research Program, Research Programs Unit, University of Helsinki, Biomedicum Helsinki, PO Box 63 (Haartmaninkatu 8), FI-00014 Helsinki, Finland; 3 Division of Functional Genomics and Systems Biology, Department of Medical Biochemistry and Biophysics, Karolinska Institutet, Scheeles väg 2, SE-17165 Stockholm, Sweden; 4 Genetic Medicine and Development, University of Geneva Medical School-CMU, 1 Rue Michel-Servet, 1211 Geneva, Switzerland; 5 Department of Molecular Medicine, Aarhus University Hospital, DK8200 Aarhus N, Denmark; 6 Department of Pathology, HUSLAB and Haartman Institute, Helsinki University Central Hospital, Haartmaninkatu 3, FI-00290 Helsinki, Finland; 7 Department of Gastrointestinal Surgery, Helsinki University Hospital, University of Helsinki, Haartmaninkatu 4, FI-00290 Helsinki, Finland; 8 Department of Surgery, Jyväskylä Central Hospital, Keskussairaalantie 19, FI-40620 Jyväskylä, Finland; 9 Department of Health Sciences, Faculty of Sport and Health Sciences, University of Jyväskylä, PO Box 35, FI-40014 Jyväskylä, Finland; 10 Department of Biochemistry, University of Cambridge, Tennis Court Road, Cambridge, CB2 1GA UK; 11 Department of Biosciences and Nutrition, Karolinska Institutet, SE-17177 Stockholm, Sweden

## Abstract

Point mutations in cancer have been extensively studied but chromosomal gains and losses have been more challenging to interpret due to their unspecific nature. Here we examine high-resolution allelic imbalance (AI) landscape in 1699 colorectal cancers, 256 of which have been whole-genome sequenced (WGSed). The imbalances pinpoint 38 genes as plausible AI targets based on previous knowledge. Unbiased CRISPR-Cas9 knockout and activation screens identified in total 79 genes within AI peaks regulating cell growth. Genetic and functional data implicate loss of *TP53* as a sufficient driver of AI. The WGS highlights an influence of copy number aberrations on the rate of detected somatic point mutations. Importantly, the data reveal several associations between AI target genes, suggesting a role for a network of lineage-determining transcription factors in colorectal tumorigenesis. Overall, the results unravel the contribution of AI in colorectal cancer and provide a plausible explanation why so few genes are commonly affected by point mutations in cancers.

## Abstract (web summary)

In this study the authors examine the allelic imbalance (AI) landscape of colorectal cancer, reporting loss of *TP53* as a driver of AI. They use CRISPR-Cas9 screens to identify 79 genes (within AI regions) regulating cell growth and identify a network of transcription factors that may contribute to colorectal tumorigenesis.

## Introduction

The so-called multistage model of carcinogenesis emerged in the 1950s with age-dependent mortality data suggesting approximately six to seven rate-limiting steps leading to oncogenesis^1,2^. Ever since then much work has revolved around the number and identity of somatic driver events required for genesis of malignancy. In particular, stepwise mutations occurring in stem cells (SCs) or progenitor cells are currently believed to be of key importance^3^.

Much of the progress in cancer research has, in recent years, emanated from high-throughput sequencing efforts that have revealed a plethora of validated and putative driver mutations across tumor types^4,5^. One unexpected lesson learned early in the process was that frequently mutated driver genes are few^6^.

While the knowledge on point mutations in tumorigenesis has expanded rapidly, the research community has somewhat neglected the contribution of allelic imbalances (AI)—losses and gains of genetic material often comprising whole chromosomes or chromosome arms^6^. Indeed, the first genomic analyses on solid tumor DNA focused on detection of copy number alterations in genomes of various cancers^7^. In colorectal cancer (CRC), genome-wide copy number analyses have uncovered well-established gross changes which include for example loss of the loci harboring tumor suppressors *TP53*, *PTEN*, *SMAD4* and *APC*, and gain of *MYC*^8^. The main obstacle in AI analysis has been the somewhat limited ability of the approach to pinpoint the main targets of larger copy number alterations that often span whole chromosome arms and thousands of genes similarly lost or gained. The difficulty is in part inherent, as it is conceivable that a single large amplification or deletion can sometimes have multiple targets which may be difficult to distinguish with classical genetic tools^8,9^. As a consequence, the contribution of gene copy number changes in cancer has remained elusive when compared to point mutations. We expect that current tools, when applied to a sufficiently large number of lesions, provide a much improved view on AI changes in CRC and thus enable identification of primary targets of gains and losses at single gene resolution.

This work provides a high-resolution data set of 1699 CRC tumor/normal pairs pinpointing 38 genes as probable AI targets based on previous literature, as well as insight into general features of AI such as high relative prevalence of fragile site deletions in microsatellite unstable (MSI) tumors and frequent formation of isochromosome i(8q) resulting in gain of the *MYC* oncogene. A long-term cell culture assay of normal and *TP53* knockout cell lines demonstrated sufficiency of this common tumor suppressor deletion for generation of large scale AI in vivo. We further studied the hundreds of candidate AI target genes by replicating the AI analysis, demonstrating the direct effects of AI on gene expression in 259 additional CRCs^10^ and by testing their effect on proliferation in CRISPR/Cas9 and CRISPRa/dCas9 CRC cell line screens. The relationship of AI and point mutations was studied by whole-genome sequencing (WGS) 256 of the tumor/normal pairs. This revealed a subtle effect of DNA copy number on the somatic mutation detection rate. Importantly, AI events clearly outnumbered somatic point mutations even in known cancer genes. Finally, we examined correlations between the copy number aberrations at those 37 peaks that harbored curated cancer genes and studied their functional genomics with ChIP-nexus/exo, siRNA and RNA-seq assays. This data revealed a network of lineage determining transcription factors (TF) converging in activation of the *MYC* oncogene.

## Results

### High-resolution allelic imbalance analysis reveals CRC drivers

We studied AI in a set of 1699 CRC tumor/normal DNA pairs extracted from fresh-frozen tissue or blood. In cancer DNA, we evaluated AI at loci found to be heterozygous in the corresponding normal sample—2.5 million single nucleotide polymorphisms (SNPs) measured for B-allele frequency (BAF)—and classified the, often large, continuous segments of equally imbalanced SNPs into losses and gains based on mean log R ratio (LRR)^11^. The two alleles at the heterozygous loci provide a perfect control for allelic relative copy number, resulting in much higher sensitivity for detecting chromosomal changes as compared to analysis based on LRR alone.

**Fig. 1:**
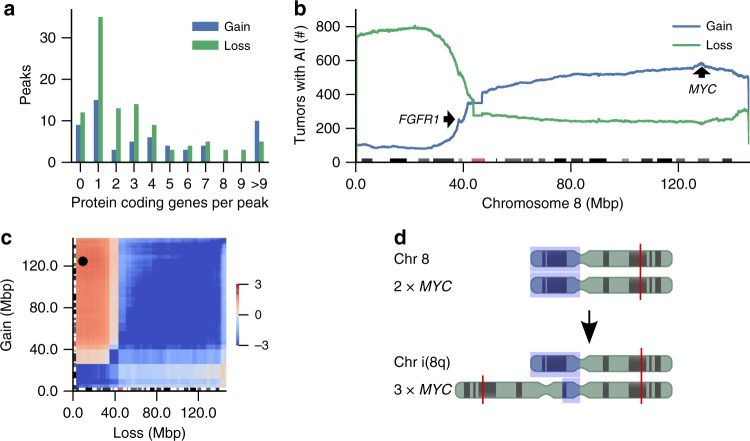
AI peaks highlight target genes with gene level resolution. **a** Number of allelic imbalance (AI) peaks with given number of intersecting protein coding genes. **b** The number of tumors showing AI along chromosome 8. Loss of heterozygosity depicted in green, gain of genetic material in blue. Cytobands are annotated at the bottom. Genes *FGFR1* and *MYC* are highlighted. **c** Log_2_ odds ratio (OR) of a sample having gain and loss on different parts of chromosome 8. Pair of sites with maximal OR = 4.3 highlighted. **d** Schematic figure of isochromosome formation. 8p arm highlighted in blue and *MYC* locus in red

We separately counted the tumors with either allelic gain or loss on every locus of the genome (“loss” refers here also to copy number neutral loss of heterozygosity (LOH)). The resulting graphs were smoothed in 10 breakpoint rolling windows and the local maxima in the smoothed graph were called as peaks (Fig. 1b). The peaks were scored according to their prominence (the height of the peak above the lowest contour line that surrounds it and does not contain a higher peak). In total, we identified 165 peaks whose prominence was at least 15 tumors (Supplementary Data 1). The large number of studied CRCs and the sensitive nature of allelic imbalance analysis as compared with copy number analysis provided a high spatial resolution for pinpointing target genes (Fig. 1, Supplementary Figure 1). While the losses and gains in a single tumor typically affected very large chromosomal segments, the areas of local maxima—peaks—were narrow due to the large number of tumors analyzed. Most often these contained only one protein coding gene (Fig. 1a).

The list of 106 loss and 59 gain AI peaks was curated based on previous literature in order to identify plausible candidate genes relevant for somatic selection. We found a strong candidate gene for 17 gain and 18 loss AI peaks (Table 1). These peaks contain well-known CRC genes such as *APC, TCF7L2*, and *MYC*, and also several genes with direct therapeutic relevance such as *KRAS, ERBB2, FGFR1*, and *PDGFRB*^12^. One peak included two plausible driver genes, *PDGFRB* and *CDX1* at 5q, and some had a likely target just outside the region, such as the tumor suppressor *TP53* of which the coding sequence locates 17 kb from a peak in chromosome 17p (Supplementary Figure 2).

**Table 1:** Literature curated 37 AI peak loci

**Gene**	**Location**	**AI type**	**Protein coding genes in peak**	**CRCs with AI**	**MSS CRCs with SNV or indel at target gene**	**Annotation**
*TP53*	17p	Loss	2	1076 (63%)	148 (63%)	S.E
*SMAD4*	18q	Loss	5	1054 (61%)	27 (11%)	STE
***HNF4A***	20q	Gain	5	804 (47%)	3 (1%)	STE
*APC*	5q	Loss	1	764 (45%)	179 (76%)	STE
*KLF5*	13q	Gain	1	709 (42%)	5 (2%)	STE
*USP12*	13q	Gain	0	694 (41%)	0 (0%)	.TE
***RUNX3***	1p	Loss	2	678 (40%)	3 (1%)	…
***TLR3***	4q	Loss	4	642 (38%)	7 (2%)	..E
***SOX9***	17q	Loss	0	621 (36%)	11 (4%)	STE
*MYC*	8q	Gain	1	585 (34%)	1 (0%)	STE
***BMPR1A***	10q	Loss	4	581 (34%)	3 (1%)	..E
*PTEN*	10q	Loss	5	581 (34%)	9 (4%)	STE
***FGFR3***	4p	Loss	3	551 (32%)	1 (0%)	S.E
*TCF7L2*	10q	Loss	1	548 (32%)	21 (8%)	STE
*PARK2*	6q	Loss	1	545 (33%)	8 (3%)	ST.
***NCOR2***	12q	Loss	1	495 (29%)	5 (2%)	S.E
***GPRC5A***	12p	Loss	3	495 (29%)	0 (0%)	STE
***SMARCA2***	9p	Loss	0	440 (26%)	5 (2%)	.TE
***CDKN2B***	9p	Loss	0	437 (26%)	0 (0%)	ST.
***RHOB***	2p	Loss	1	433 (26%)	0 (0%)	..E
***ACVR2A***	2q	Loss	3	430 (25%)	7 (3%)	S.E
***MLK4***	1q	Loss	2	428 (25%)	0 (0%)	S.E
***G0S2***	1q	Loss	9	408 (24%)	0 (0%)	S..
***CTCF***	16q	Loss	13	387 (23%)	3 (1%)	S.E
*FGFR1*	8p	Gain	2	254 (15%)	6 (2%)	S..
***CCND2***	12p	Gain	1	186 (11%)	0 (0%)	STE
*KRAS*	12p	Gain	4	179 (11%)	121 (51%)	..E
*IGF2*	11p	Gain	4	126 (7%)	0 (0%)	STE
***BPTF***	17q	Gain	5	110 (6%)	9 (3%)	STE
***NOTCH1***	9q	Gain	4	101 (6%)	4 (1%)	..E
*ERBB2*	17q	Gain	14	99 (6%)	10 (4%)	STE
***SATB2***	2q	Gain	1	83 (5%)	4 (2%)	.TE
***PDGFRB***, ***CDX1***	5q	Gain	6	69 (4%)	5 (2%)	…, ..E
***ITGB1***	10p	Gain	3	56 (3%)	0 (0%)	S.E
***FOXA1***	14q	Gain	4	48 (3%)	1 (0%)	S.E
***TNFAIP2***	14q	Gain	11	36 (2%)	1 (0%)	…
***DGCR8***	22q	Gain	11	32 (2%)	0 (0%)	..E

If the number of protein coding genes in a smoothened peak area was zero, the two flanking genes were considered as candidate targets. Gene names are in bold if they have not been previously reported as high-resolution (identified area containing 10 or fewer genes) AI target in CRC. Type of gross change at the target is indicated as “Loss” or “Gain”. The number of tumors with AI, including the homozygous losses at PTEN and SMAD4, are shown for 1699 CRCs. The number of tumors with somatic nonsynonymous SNVs and indels in the presumed AI target gene is given for the subset of 234 MSS tumors that underwent WGS. Peaks are annotated by re-discovery in the two lower-resolution datasets, 230 Danish colorectal cancers (S) and TCGA COADREAD (*n* = 573) (T), and significant expression change (FDR < 10%) (E). Citations in Supplementary Data 2

Additionally, we called balanced copy number aberrations to detect areas of clonal homozygous loss. Compatible with previous reports^9^, these were few. Recurrent homozygous losses were detected only at chromosomal fragile sites, and at the tumor suppressors *PTEN* and *SMAD4*. Thus, altogether 38 genes in 37 peaks were highlighted in our analysis; 36 by AI and 2 by homozygous loss.

The three most common loci of loss in the whole data set were *TP53* in 1072 or 63% of 1699 cancers, *SMAD4* (1040 or 61%) and 8p21.3 (806 or 47%) (Table 1). The three most frequently gained loci in the whole data set were *HNF4A* in 803 or 47% of 1699 cancers, *KLF5* (709 or 42%) and *MYC* (585 or 34%) (Fig. 1b), all of which have previously been implicated as amplification targets in CRC^8,13,14^. Our data provide high-resolution genetic validation of these genes as frequent drivers of CRC.

### MSI CRCs are chromosomally stable, except for fragile sites

Microsatellite stable (MSS) CRCs harbored 3.9-fold more allelic imbalance (by basepairs covered) than MSI CRCs. MSS cancers also showed strong preference of *TP53* and *SMAD4* losses (genome-wide-corrected logistic model, *p* < 10^−5^), whereas aberrations in MSI CRCs occurred preferably at common fragile sites *FHIT* (Logistic model, *p* < 10^−5^), *RBFOX1* (*p* < 10^−5^), *WWOX* (*p* < 0.02), and *MACROD2* (*p* < 0.05) (Supplementary Table 1).

### 8p is commonly lost through formation of isochromosome i(8q)

Loss of chromosome arm 8p is the third most common AI alteration in our data, occurring in almost half (47%) of the cancers examined. Frequent loss of chromosome 8p has been firmly linked to colorectal tumorigenesis for decades^15^, but the identity of the possible target genes has remained elusive. Genome-wide inspection of correlations between gains and losses within chromosomes revealed that 8p loss and *MYC* gain (in 8q) co-occurred in the same samples (OR = 3.9, *p* < 3.3 × 10^−29^, Fisher’s exact test, Fig. 1c). Furthermore, analysis of WGS data revealed frequent chromosome 8 AI and copy number changes co-occurring with sequence inversion in the pericentric region in the p-arm of the chromosome. This signals formation of isochromosome i(8q) with two centromeres and q arms and a small, duplicated and inverted segment of the p-arm (Fig. 1d)^16^. In total 409 tumors (24%) appear to have undergone isochromosome i(8q) formation (Methods), gaining a copy of 8q (including oncogene *MYC*) and losing a copy of 8p in a single chromosomal event.

### Mutant *KRAS* locus is commonly low level gained in CRC

One of the curated gain targets, *KRAS*, is often activated by somatic mutations and there have been reports of exclusivity of *KRAS* amplification and mutation^17^. We Sanger sequenced the *KRAS* gene in 1447 of our samples and correlated the point mutation and the copy number statuses at the *KRAS* locus in these tumors. While 31% (279/893) of the samples with no AI at *KRAS* had a somatic point mutation, 72% (129/178) of the samples with copy number gain AI at *KRAS* did have a mutation. The raw LRR values at the *KRAS* locus reveal also a small subset of wild type samples with strong amplification (Supplementary Figure 3).

### Gene expression levels reflect copy numbers

AI analysis was repeated in an additional set of 259 colorectal tumors, and previously generated RNA-seq data^10^ from these tumors was analyzed for a potential association between AI copy number and gene expression (see Methods). As expected, the majority of the curated AI peak genes displayed significant association between the copy-number variation at the AI peak and gene expression (Wald test, Benjamini–Hochberg FDR < 10%, Table 1, Supplementary Figure 4, Supplementary Table 2 and Supplementary Data 3).

Furthermore, to connect the functional effects of chromosomal changes to overall patient survival, we analyzed whether genes whose expression is associated with a favorable or unfavorable prognosis^18^ are on average gained or lost in our data. Interestingly, the genes whose expression is associated with favorable prognosis are lost more often than genes associated with unfavorable prognosis (*p* < 10^−24^ Mann–Whitney *U*-test). Conversely, the genes associated with unfavorable prognosis are gained more often than genes associated with favorable prognosis (*p* < 10^−11^, Mann–Whitney *U*-test). Although the list of favorable/unfavorable genes is not controlled for copy-number and thus contains abundance of passengers, the clinical effect is also reflected in our data as a slight survival advantage for patients with low amount of AI (*p* = 0.02, Kaplan–Meier log-rank test, Supplementary Figure 5).

### High copy number regions host more single base substitutions

**Fig. 2:**
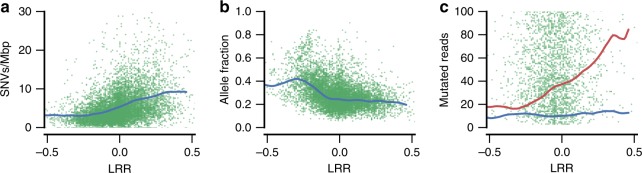
Somatic copy number with respect to single-base substitutions. **a** Increasing number of single base variant calls per megabase of reference sequence as a function of copy number (LRR, Log R Ratio). **b** Nearly uniform mean proportion of mutated reads as a function of LRR when at least a normal number of DNA copies are present. **c** Nearly uniform number of mutated reads observed per SNV as a function of LRR. Mean number of reference allele reads for comparison (red, lowess fit). Each (green) point stands for an AI segment (**a**) or a point mutation (**b**, **c**) on strictly callable^38^ genome segment with called AI in MSS tumors. Blue lowess fit curves are provided as a visual guide. LRR axes cover more than 99.9% of AI regions

Next we analyzed the relationship of somatic copy number changes and single-nucleotide variants (SNV) that were detected in the same samples. WGS data were generated from a subset of the 1699 CRCs consisting of 234 microsatellite stable (MSS) tumors and their respective normal tissues. Analysis of the regulatory genome (noncoding) mutations of 213 of these has been published previously^19^. We observed that the density of SNVs increase with the LRR copy number measure (Fig. 2a). The clonality of the SNVs is nearly uniform as long as there is more than one chromosome copy present in the tumor (Fig. 2b). Also the median count of the mutated reads on a given SNV is mostly independent of copy number (Fig. 2c), suggesting that the increased rate of SNVs is not only due to improved detection sensitivity but displays increased mutation density at high copy number regions. These data are compatible with a neutral model of somatic mutations where each chromosomal copy obtains somatic mutations independently with constant rate per replicated base pair.

Frequent large scale copy number aberrations cast doubt on some tools for identifying cancer genes if the underlying copy number is not accounted for as a source of background mutation rate variation^20^. For example MutSig2CV p-values for gene mutation significance in the TCGA colorectal cancer project have a negative correlation with the mean LRR in the samples in this study (Supplementary Figure 6)^4^. Similarly, driver *p*-values from 20/20+ analysis of pancancer data are slightly smaller in high rather than low copy number regions^20^ (Supplementary Figure 7). The copy number bias does not seem to similarly affect OncodriveFML^21^, which models mutation likelihoods in local genomic context (Supplementary Figure 8).

### Most cancer genes have higher prevalence of AI than SNVs

**Fig. 3:**
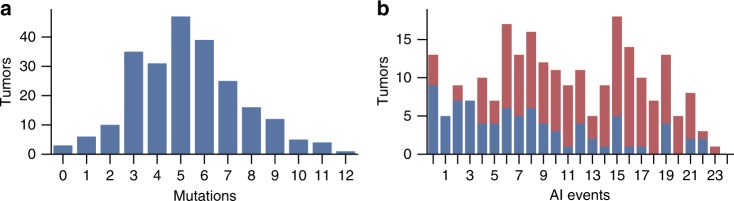
Coding point mutations and allelic imbalance in 234 MSS CRCs. **a** Number of somatic point mutations per tumor in 74 significantly mutated and/or known CRC-associated genes. Total of 1241 mutations; median 5 per tumor. **b** Number of AI events in curated peaks per tumor. Total of 2501 AI events in 37 peaks stratified by the *TP53* mutation status (WT: blue, 6.5 events/tumor; *TP53* mutated: red, 12.5 events/tumor; Mann–Whitney rank test *p* = 3 × 10^−8^). Only gain (loss) events counted for gain (loss) type peaks

Using OncodriveFML^21^, we identified a total of 42 genes targeted by significant numbers of somatic point mutations in the set of 234 whole genome sequenced MSS tumors (OncodriveFML, Benjamini-Hochberg FDR < 10%, Supplementary Table 3, Supplementary Figure 9). Analysis of the mutation burden in these 42 genes, and 42 genes associated with CRC in the COSMIC cancer gene census (total of 74 unique genes), showed that the point mutation rate in MSS tumors rivaled the rate of respective chromosomal aberrations in only *APC*, *KRAS*, and *TP53* (Supplementary Figure 10). The median value of cancer gene point mutations per tumor was 5 (Fig. 3a, Supplementary Table 4), whereas a median of 10 curated peak loci displayed expected type (gain for oncogenes in gain type peaks, loss for tumor suppressors in loss type peaks) of AI in each tumor (Fig. 3b). These results highlight the major role of AI in CRC even considering that a subset of AI events hitting cancer genes are likely to be random.

### TP53 loss enables allelic imbalance in normal cells

Evaluating the clonality of the curated loss peaks with the tumor purity corrected B-allele frequency (cBAF) revealed that *TP53* had the highest mean clonality of all peaks, followed by *APC, SMAD4*, and *SOX9* (Supplementary Figure 11). This is compatible with relatively uniform presence of these gene defects in cells forming the surgically removed tumor. We probed the role of *TP53* loss in downstream mutational processes as this gene has been widely accepted as the “guardian of the genome”^22^. We cultured wild type normal human RPE1 cells and three clonal RPE1 *TP53*-null lines^23^ for 6 months in parallel. In whole-genome sequencing of the four lines the *TP53*-null cells displayed a weak, not statistically significant 23–46% increase in single-nucleotide variants (SNVs) (*p* = 0.08, negative binomial, Supplementary Table 5, Supplementary Figure 12), compared to the *TP53* wild-type line. The *TP53*-wild type control and one of the mutant lines (KO3) did not show any novel allelic imbalances. However, two of the *TP53*-null lines displayed striking allelic imbalances in two (KO17) and twelve (KO6) whole chromosomes. RNA-seq of the cultured cells revealed high median expression of genes on the balanced chromosomes of KO6, suggesting a biallelic genome duplication followed by a series of chromosomal losses, producing a cell with three (imbalanced) or four (balanced) copies of each chromosome. The chromosomal aberrations were large, with no trace of chromothripsis.

To further validate the high rate and diversity of AI events in *TP53*-null cells, we applied SNP array genotyping to two clonal lines (KO6.2 and KO17.3) separated from KO6 and KO17 by 2 weeks of culture. These lines showed additional AI on two (KO17.3) and one (KO6.2) chromosomes. A similar effect was detected in the primary tumor samples (Fig. 3b and Supplementary Figure 13). Taken together these data demonstrate that TP53 deficiency is sufficient for the cell to tolerate aberrant gene dosage and thus facilitates accumulation of tumorigenic copy number changes.

### CRISPR screens reveal role of AI genes on cancer cell growth

In order to identify the AI target genes within the 165 AI peaks we systematically probed the 927 protein coding genes residing in the identified peak areas—including six flanking genes for each peak—for their potential role in CRC cell proliferation. This was done by 33-day long pooled CRISPR/Cas9 knockout^24^ and CRISPRa/Cas9d activation screens^25^ in three CRC cell lines, GP5d, COLO320DM and CaCO2 (Supplementary Figure 14, Supplementary Data 4 and 5). These assays detect genes whose loss or overexpression increases or decreases growth of tumor cells in culture.

In the loss-of-function CRISPR/Cas9 screen, several genes with a known CRC regulatory role such as *MYC, KLF5, CCND2*, *BPTF*, *TCF7L2*, *CTCF*, and *SMAD4* together with 58 other genes, were identified as required for cell growth in at least one of the three cell lines (Robust Rank Aggregation, FDR < 0.1). Copy number status of the cell line can have a strong influence on the CRISPR/Cas9 results^26^ but in our case the effect was limited to at most five genes for which the loss-of-function phenotype was detected only in a cell line where the gene is located in a copy-number-gained region (Supplementary Methods, Supplementary Data 4). Among the genes required for growth was also *BPTF*, a cancer gene less studied in CRC. As expected, growth inhibitory genes (loss promotes growth) were detected more rarely; only *PTEN* was identified in two cell lines, 5 other genes in one. A growth phenotype was detected in at least one of the cell lines for 71 genes located in 45 peak areas in total. These genes serve as additional AI target candidates.

The results of our loss of function screen were highly consistent with the results obtained in other CRC cell lines screened genome-wide but using fewer guide RNAs per gene by the Cancer Dependency Map Project^27^. From the 795 genes in AI peak areas that were measured by both, the twelve genes whose knock-out had the largest negative effect on growth across CRC lines in their screen were all identified as being required for cell growth in at least one cell line in our screen (Hypergeometric *p* < 10^−13^, Supplementary Figure 15, Supplementary Data 4). Conversely, the 61 genes identified in our screen as required for cell growth for at least one cell line had a median rank of 62 in their screen within the 795 shared genes (Mann-Whitney *U*, *p* < 10^−23^). More generally, the genome-wide results highlight the particular importance of the AI target genes for CRC: of the 17,670 genes measured, six of the top ten genes that the CRC cell lines were particularly dependent on (versus other cancer cell lines) were in the AI peaks: *TCF7L2, SATB2, KRAS, KLF5, CCND2*, and *DBF4* (Fisher’s exact test *p* < 2 × 10^−7^).

The gain of function CRISPRa/dCAS9 experiment found a growth phenotype for 9 genes (Supplementary Data 5). Activation of *PDGFRB*, *PIGQ*, and *MYC* (and as a likely bystander its adjacent gene *CASC11*), increased cell growth while activation of *SOX9, IKZF3, IRF6*, *RASAL2,* and *KRAS* inhibited growth. Notably, only COLO320DM growth was promoted by activation of MYC, which is already >60× amplified in its genome and was the only hit in the activation screen with copy number alteration in the tested cell line. Also worth noting, GP5d growth was inhibited by activation of KRAS-G12D, while KRAS activation in wild type cell lines (CaCO2, COLO320DM) did not have a detectable effect.

The only shared hit between knockout and activation screens was *MYC*, further highlighting its central role in CRC cell proliferation.

### TFs in AI peaks co-operate toward *MYC* activation

**Fig. 4:**
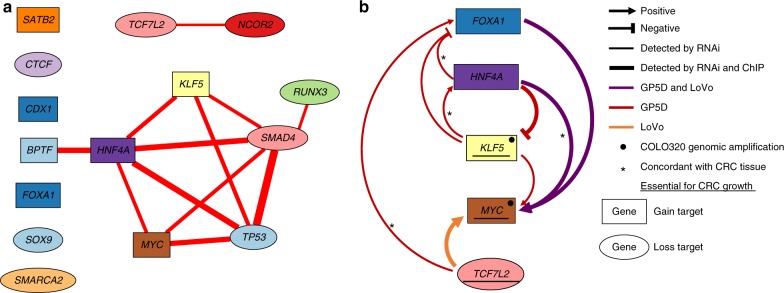
Functional genomics scrutiny of associations between curated TF hits. **a** Curated AI peaks that contained key TFs associated with each other. Oval and rectangle nodes represent loss and gain peaks, respectively. An edge is drawn between nodes if genome wide corrected association *p*-value < 0.001 (logistic regression). Width of edge is proportional to effect size. All edges represent positive association. **b** Regulatory circuitry of the transcription factor targets of AI. siRNA silencing followed by RNA-seq were used to detect positive and negative regulatory relations between TFs with FDR < 0.05 (Wald test). Red edges detected in GP5d cell line, orange in LoVo, purple in both. Asterisks depict concordant expression relation between the pair of TFs (FDR < 0.05) in the regression analysis of RNA-seq data from 259 colorectal tumors as compared to siRNA experiments (details in Supplementary Methods). Thick lines show potentially direct regulation based on the presence of the corresponding ChIP-nexus/exo peaks. Note that the cell lines tested displayed different regulatory states of the network, but the *MYC* gene was essential in all, based on the CRISPR/Cas9 screen, suggesting that individual tumors utilize different upstream mechanisms to drive cell growth through MYC^39^

We examined interchromosomal correlations between the copy number aberrations at the 37 peaks with curated genes. All significant associations (Logistic regression, Bonferroni-corrected *p* < 0.05. Supplementary Methods) observed are depicted in Supplementary Figure 16. A particularly striking finding is a clique of transcription factors that tend to display AI in the same samples (Fig. 4a). To find possible loops of aberrant regulation within the clique, we studied the target genes and DNA binding patterns of FOXA1, HNF4A, KLF5, MYC, and TCF7L2 transcription factors (Supplementary Figure 17 and Tables 6-9). We performed ChIP-nexus/exo binding assays and siRNA knockdown followed by RNA-seq in three (LoVo, GP5d, COLO320DM) colon cancer cell lines. Direct target genes of TFs were searched from the same topologically associated domains^28^ in which ChIP-nexus/exo peaks were identified. To detect up- or downregulated genes after siRNA knockout of the TFs, we used sleuth^29^ to compare RNA-seq data from perturbed experiments to the control (non-targeting siRNA) experiment. Furthermore, expression of each TF was studied in 259 colorectal tumor RNA-seq data with a regression model including LRR of the target TF, expression levels of the other four TFs and tumor purity as predictors. These experiments, summarized in Fig. 4b, provided evidence for formation of a regulatory loop between some of the key factors that converged on regulation of the *MYC* oncogene (Supplementary Data 6 and 7 and Supplementary Table 10).

## Discussion

We hypothesized that the principal targets of both chromosomal gains and losses could be determined at a gene level resolution through genetic analysis if enough data for a specific tumor type was available. While our current effort was extensive, improved resolution of events—in particular those that are relatively rare (Table 1)—can be anticipated as the amount of high-quality data accumulates and reaches thousands of samples. Our data precisely identify 38 plausible targets in total, 18 for gain and 20 for loss. In our study, the “loss” category also includes copy number neutral loss; such a change can equally flag selection of either hypoactive or hyperactive alleles. To our knowledge, 26 of the 38 genes have not been reported as high-resolution targets for AI in CRC (Table 1). In low-resolution studies, a very large number of genes have been reported to be lost/gained but the highly unspecific nature of such information has not always served well the efforts to understand the disease. As expected, MSI CRCs were in general much more chromosomally stable than MSS tumors. However, deletions at fragile sites were as frequent in MSI as in MSS CRCs. The mechanisms causing chromosomal changes at fragile sites appear to be shared in MSI and MSS CRCs, and are probably different from the forces driving chromosomal instability elsewhere.

The most common AI event with no obvious candidate target was loss at 8p21.3, affecting 806 (47%) of 1699 cancers examined. Importantly, we concluded that loss of the 8p-arm typically occurred through a single chromosome 8 event leading to 8q isochromosomes and concomitant gain of *MYC* and loss of 8p as seen from the WGS data. Thus frequent loss of 8p in CRC may at least in part be a passenger event, driven by the strong positive selective value of *MYC* gain.

AI at the *KRAS* locus was strongly associated with presence of an activating mutation, providing an example of co-operation between chromosomal gross changes and base level events. High-level amplifications were only seen in KRAS wild-type lesions, compatible with possible adverse cellular effects of too much KRAS activity. Evidence for this was also seen in the CRISPRa screen where further activation of mutant *KRAS* was found to be detrimental. The genetic and CRISPRa data are consistent with three different routes of KRAS activation, either through *KRAS* point mutation with or without gain of the mutant allele, or through strong amplification of wild-type *KRAS*. Whether the different states affect response to treatment is worth further study.

When analysing the relationship between somatic copy number changes and SNVs, we observed an increase in the density of SNVs with increasing copy number (Fig. 2a). Each chromosomal copy appears to obtain somatic mutations independently with constant per base pair rate. The effect of high copy number regions accumulating increased numbers of mutation calls causes a bias in many NGS analysis approaches, and should be taken into account in the future.

Our data draw a picture where allelic imbalance (enabled e.g. by *TP53* defect) introduces a high number of aberrations in key genes involved in control of lineage development and cellular growth resulting in poor patient survival. The results highlight the importance of aberrations in intestinal stem cell homeostasis in colorectal carcinogenesis as several key genes encoding factors known to be involved in its maintenance were located at frequently targeted regions: *HNF4A, APC, RUNX3, NOTCH1, KLF5, SOX9, BMPR1A, TCF7L2*, and *CDX1*^30,31^. One emerging hypothesis from our data is that AI contributes to the establishment of positive and dynamic regulatory loops between key transcription factors; loops that are selected for due to resulting aberrant cellular homeostasis that either increases the risk of secondary tumorigenic events, or directly maintains the cancer phenotype. The networks are connected to the cell growth machinery via the *MYC* oncogene, which itself is an AI target and essential for growth of all of the cell lines analyzed here. Based on the differential requirement of some of the AI target genes for cell growth in the CRISPR/Cas9 screen, and the fact that different tumors often harbor different AI events and mutations, it appears that individual tumors achieve a similar locked gene regulatory state that drives *MYC* expression and cell growth via different upstream AI events, somatic mutations and/or amplification of *MYC* itself. AI-driven regulatory mechanisms may also be adjustable to the changing requirements of neoplastic progression. For example, frequent losses at *SOX9* (seen in more than one third of all samples, Table 1) and the observed growth inhibition in the CRISPRa assay are compatible with selection of reduced *SOX9* expression while the gene was consistently overexpressed in the RNA-seq data from the surgically removed full-blown cancers in both cases with AI, as well as those without (Supplementary Figure 18). Overall, the data suggest that the selection pressure on *SOX9* expression varies in direction during different stages of tumor development.

The significant potential of AI in developing genetic aberrations (Fig. 3) provides a plausible explanation why so few genes are commonly mutated in most cancers. Not many genes are likely to be so important for malignant transformation that their fixed point mutations are frequently selected for. Instead, during tumorigenesis, preference can be envisioned for gross chromosomal changes affecting expression levels of the main targets and multiple other genes through a single event, while still retaining flexibility to the changing needs of the neoplastic process. Given the prevalence of the AI events, robust knowledge of the main AI targets and the contexts in which they are active in tumorigenesis is essential for understanding cancer. Our data warrant serious consideration when designing functional experiments modeling different aspects of cancer gene interplay, as well as examining responses to therapy. The next straightforward step is to robustly identify the main targets of gene dosage aberrations across all tumor types. Compilation of such knowledge should be an important near-term goal for cancer genomics research. The current work demonstrates how accumulation of dense genetic data from thousands of lesions of a given type, and paired normal tissue, produces accurate catalogs of highly relevant genes to be coherently evaluated as important pieces in the puzzle of cancer.

## Methods

### Samples

The study has been reviewed and approved by the Ethics Committee of the Hospital District of Helsinki and Uusimaa (HUS). Signed informed consent or authorization from the National Supervisory Authority for Welfare and Health has been obtained for all Finnish sample materials used. The use of the Danish tissue samples was approved by the Central Denmark Region Committees on Biomedical Research Ethics. Informed written consent was provided by all Danish participants.

### Genotyping and AI analysis

The tumor and respective normal DNA samples were genotyped with Infinium Omni2.5-8 (Illumina Inc.) array at Estonian Genome Center. The B-Allele Frequencies and Log-R ratios were extracted with Illumina Genome Studio software and the allelic imbalance regions were calculated for all samples using BAFsegmentation^11^ with default parameters. AI count graph was generated by counting tumors with AI on particular locus using bedtools genomecov^32^ separately for gains and losses. The AI count graph was smoothed with max function in a window of 10 consecutive breakpoints. Peaks were called as local maxima of the smoothed AI count graph. Peaks were ranked according to their topographic prominence, the height of the peak above the lowest contour line that surrounds it and does not contain a higher peak, in the unsmoothed AI graph. The highest peak of a chromosome has prominence equal to its height. The peaks with prominence of at least 15 were reported. A tumor sample was called as having an isochromosome if at least 80% of at least one chromosome arm is called allelic imbalance and the mean LRR on the p-arm is at least 0.2 less than on the q-arm. The 259 Danish tumor samples were genotyped and analyzed with same the methods except the germline heterozygous sites were obtained from prior genotyping^10^.

Phenotype and cross-peak associations were studied with logistic regression controlling for the sum of the lengths of AI regions on chromosomes other than chromosome of the studied peak(s).

### Whole-genome sequencing

Sequencing of 256 CRC and respective normal samples was carried out with an Illumina HiSeq 2000 and HiSeq X10 (Illumina Inc, and SciLifeLab) using a paired-end sequencing protocol. Read length was 100 bp.

### TF binding and RNA-seq

ChIP-nexus, -exo, and -seq was performed as described previously^33–35^. Experimental details and antibodies used are listed in Supplementary Methods.

LoVo, GP5d, and COLO320DM cell lines were treated in triplicates with human ON-TARGETplus siRNA smartpools (GE Dharmacon) to silence each of the 38 AI target genes individually and their gene expression was measured with RNA-seq. The expression was quantified with Kallisto^36^ and analyzed with Sleuth^29^.

### CRISPr screens

For CRISPko screen, guide RNAs (gRNAs) were designed to target the first and second exons of 1928 candidate target genes in AI regions and 198 control genes. For CRISPRa, the gRNAs target the first 200 bp upstream of each TSS as previously suggested. 2 × 2 × 12472 sgRNA oligonucleotides (96–102 nucleotides) were ordered from CustomArray for both CRISPRko and CRISPRa screens. A minimum of 40 million Cas9 or dCas9 expressing GP5d, COLO320DM or CaCO2 cells were transduced (MOI 1) with lentivirus pool containing sgRNA libraries. First timepoint sub-culturing was performed 4 days after transduction. Cells were grown for 33 days after transduction with sub-culturing being performed every 4–6 days. Screens in all cell lines were performed as duplicates.

The gene level significance in the CRISPR screens was evaluated using Robust Rank Aggregation^37^

All genomic coordinates are reported in GRCh37 assembly. The custom software used is available from the authors. Further method details are available in the Supplementary Methods.

## Electronic supplementary material

Supplementary Information

Description of Additional Supplementary Files

Supplementary Data 1

Supplementary Data 2

Supplementary Data 3

Supplementary Data 4

Supplementary Data 5

Supplementary Data 6

Supplementary Data 7

Supplementary Data 8

Supplementary Data 9

Supplementary Data 10

## Data Availability

The sequence data are available from ENA accession PRJEB25645 and EGA accessions EGAS00001002966, EGAS00001003010. Per sample AI regions, CRISPr/Cas9, CRISPRa guide designs and RNAi-RNA-seq analysis results are available in Zenondo repository 10.5281/zenodo.1222172. All other remaining data are available within the Article and Supplementary Files, or available from the authors upon request.
