# Supplementary material for: Contribution of allelic imbalance to colorectal cancer
Source: Nat Commun. 2018 Sep 10;9:3664. doi: 10.1038/s41467-018-06132-1 (PMC6131244; doi:10.1038/s41467-018-06132-1)
Supplement: Supplementary file 1 — Supplementary Information [file 41467_2018_6132_MOESM1_ESM.pdf]

Supplementary Information

Palin et.al. Contribution of allelic imbalance in colorectal cancer

Contents:

- Supplementary Figures 1-18
- Supplementary Tables 1-10
- Supplementary Methods

Supplementary Figures

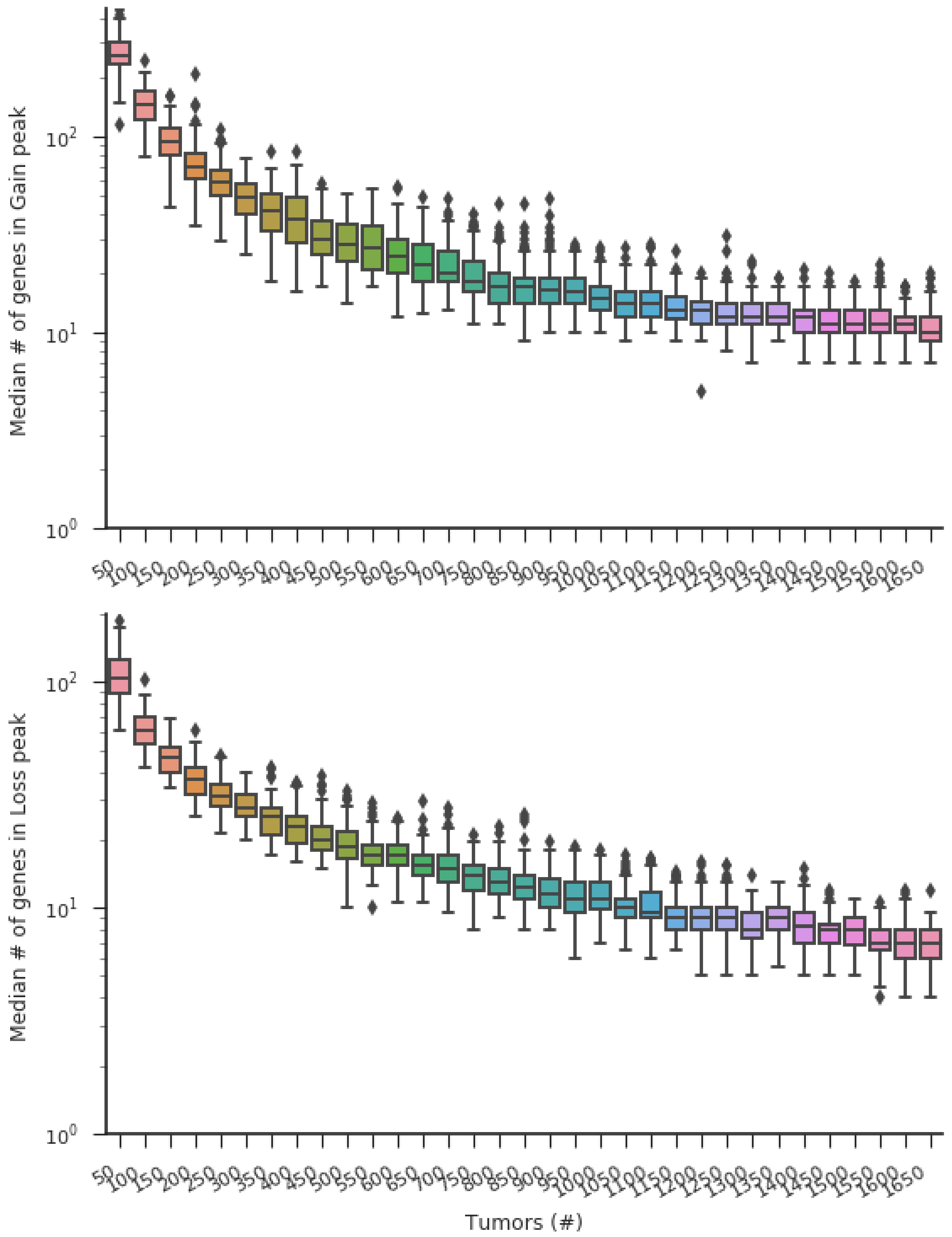

Supplementary Figure 1. Number of protein coding genes in peaks with fewer tumors analysed. Each box is from 100 independent subsamplings with replacement of original tumor data. The number of genes is calculated from area spanned by full data peak and nearest peak from subsampled data with lowest prominence threshold giving at most as many peak calls as in full data. Each box covers second and third quartiles, line is at median, high whisker extend to last datum less than 3rd quartile + 1.5*inter quartile range and low whisker to first datum greater than 1st quartile - 1.5*inter quartile range. Data outside whisker range

are plotted as separate points.

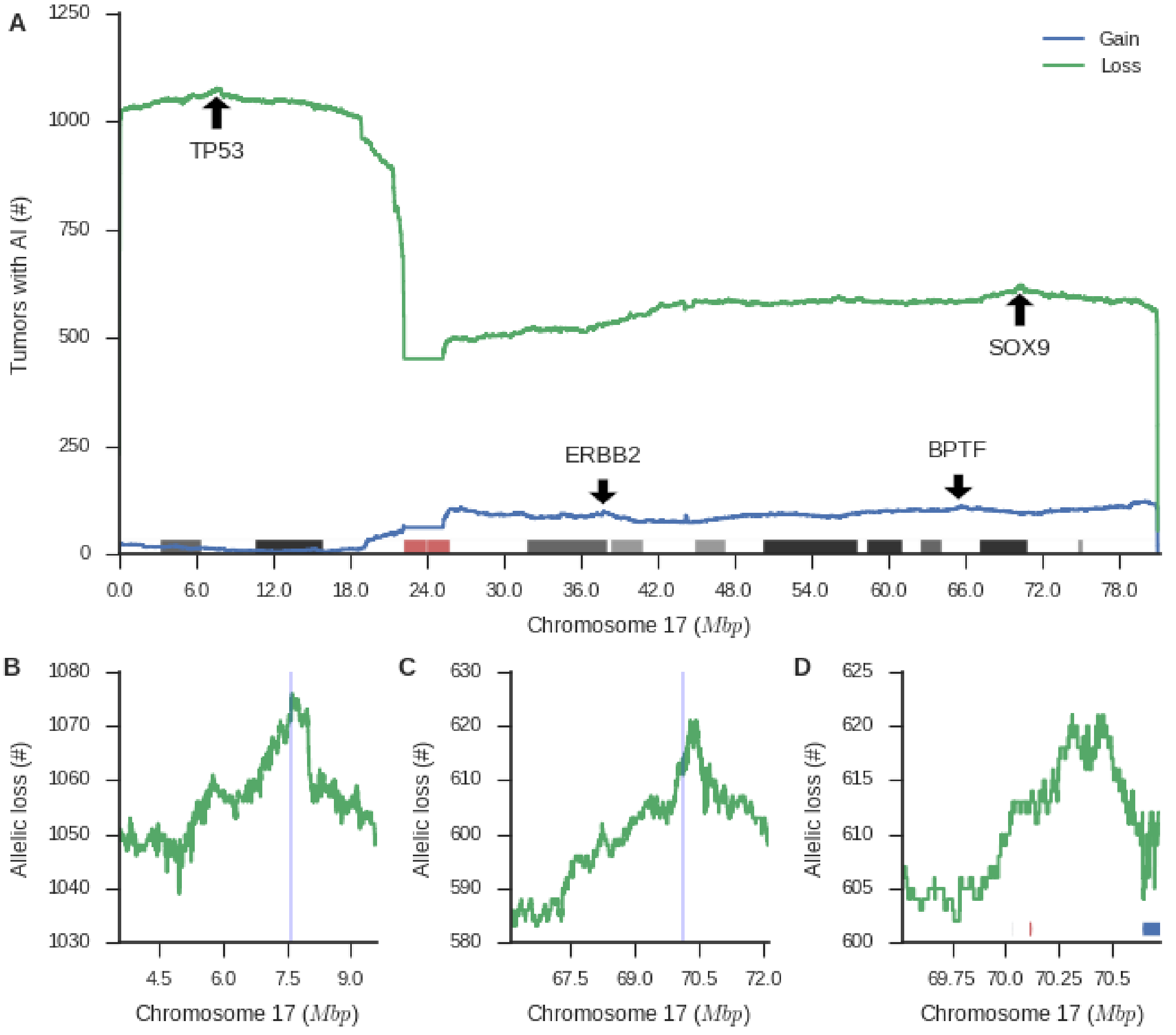

Supplementary Figure 2: A) Number of gain and LOH/loss tumors over chromosome 17 and closeup views of number of LOH/loss tumors near **B)** TP53 (gene highlighted) and **C), D)** SOX9. In **D)** protein coding genes in the region are annotated with SOX9 highlighted in red.

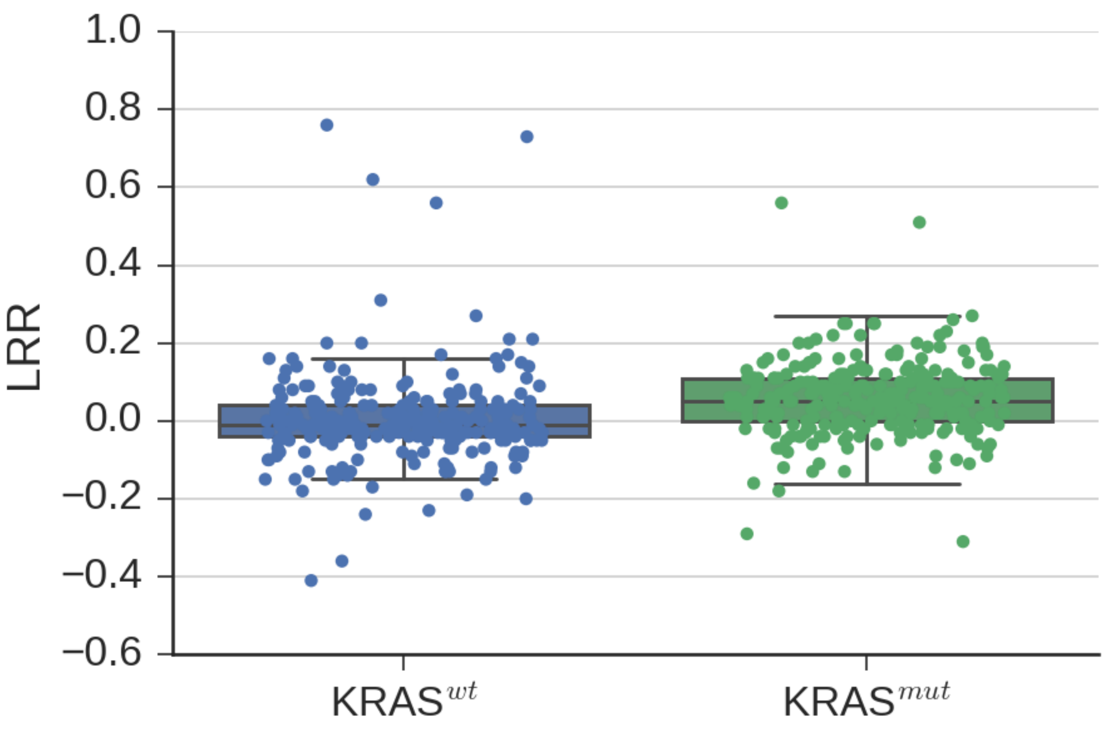

Supplementary Figure 3. Mutated KRAS is commonly amplified in colorectal cancer. Log-R ratio on y-axis. KRAS wildtype and mutant samples plotted separately.

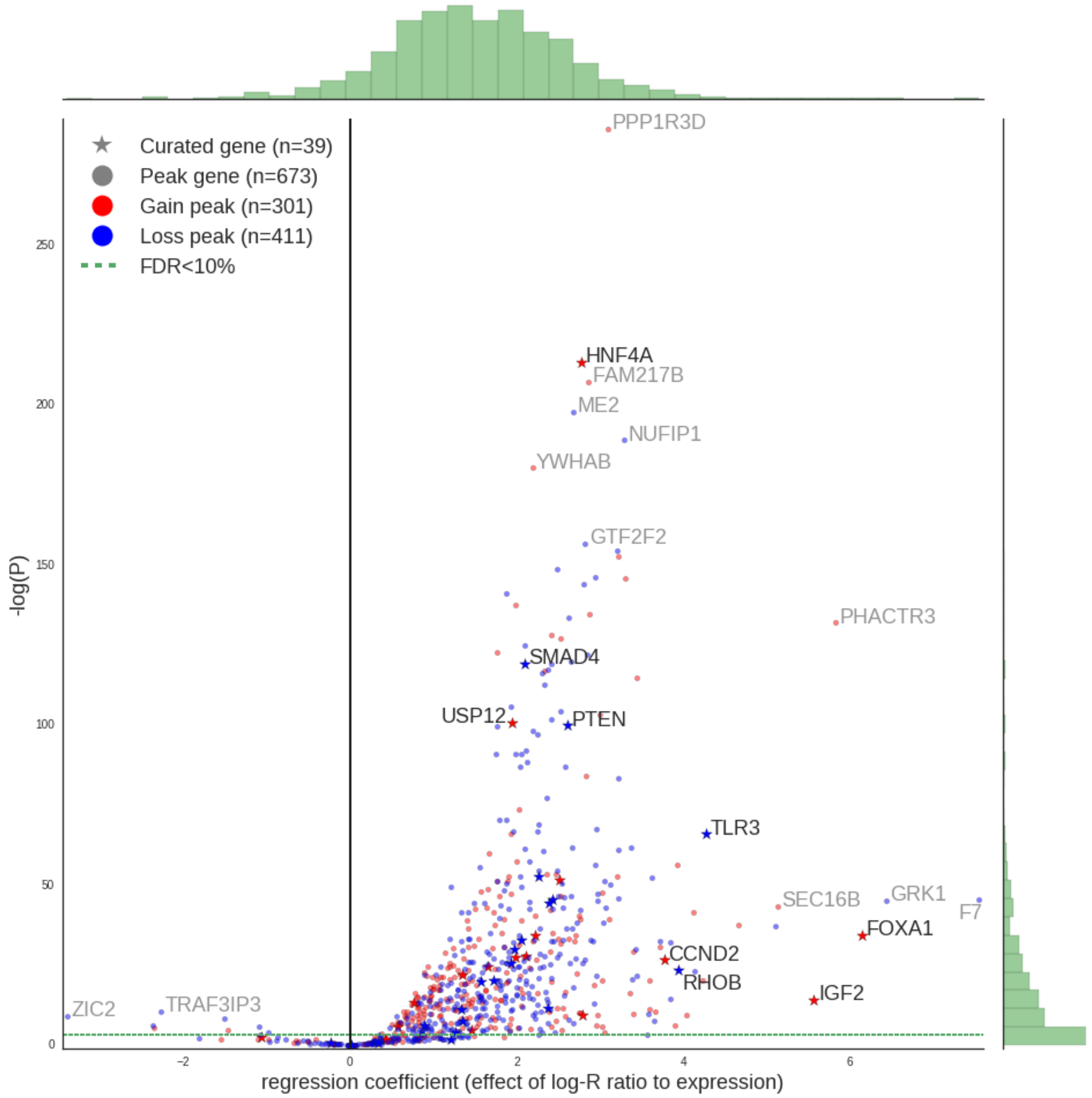

Supplementary Figure 4. Association between estimated somatic copy-number and gene expression for AI peak region genes (total 712 genes with quantifiable RNA-seq data). Positive regression coefficient (X axis) values suggest that copy-number gains increase expression and losses decrease it. Y-axis is base-2 log false discovery rate. In total 557/712 genes pass FDR<10%. Detailed numbers in Supplementary Table 2 and Supplementary Data 3.

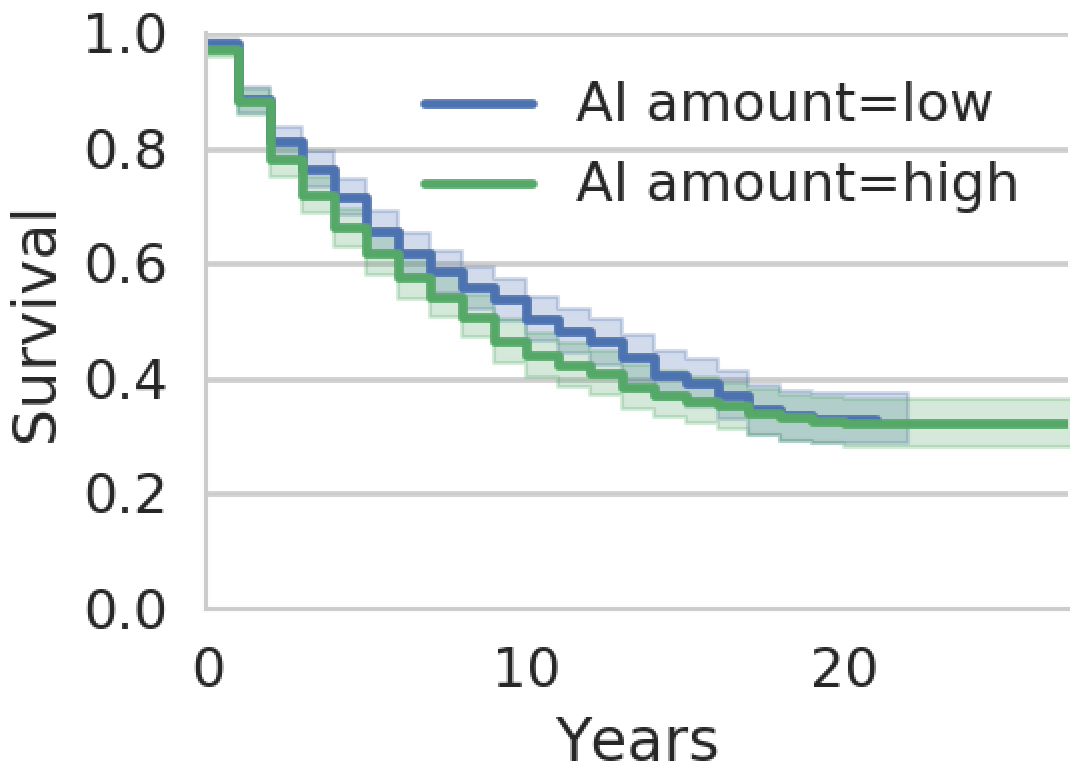

Supplementary Figure 5: Kaplan-Meier plot of overall survival for 774 CRC patients with low (<835Mbp, median over all 1699 tumors) amount of AI and 759 with high amount of AI. Log-rank p-value = 0.02

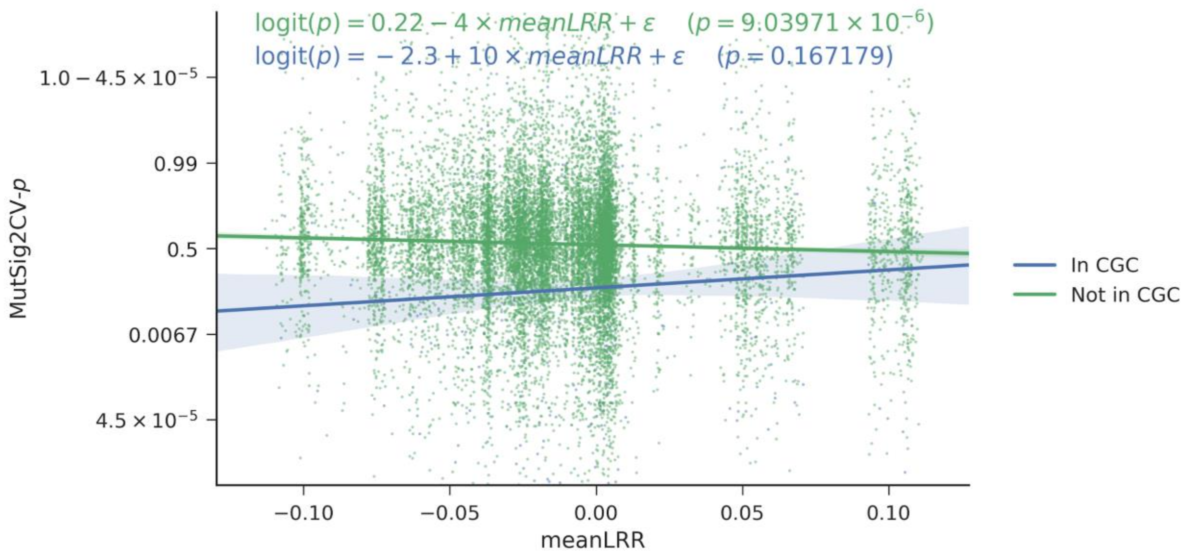

Supplementary Figure 6 Linear dependency of MutSig2CV logit(p-value) on mean LRR of the gene in COADREAD dataset. LRR values computed from 1699 samples in this study. MutSig2CV p-values from 489 samples of TCGA colon- and rectal adenocarcinoma project, downloaded from TCGA GDAC doi:10.7908/C1KW5FDZ. Genes with at least one observed somatic mutation included. Linear least squares model to logit(p) values is fitted separately to genes in/not in COSMIC Cancer Gene Census.

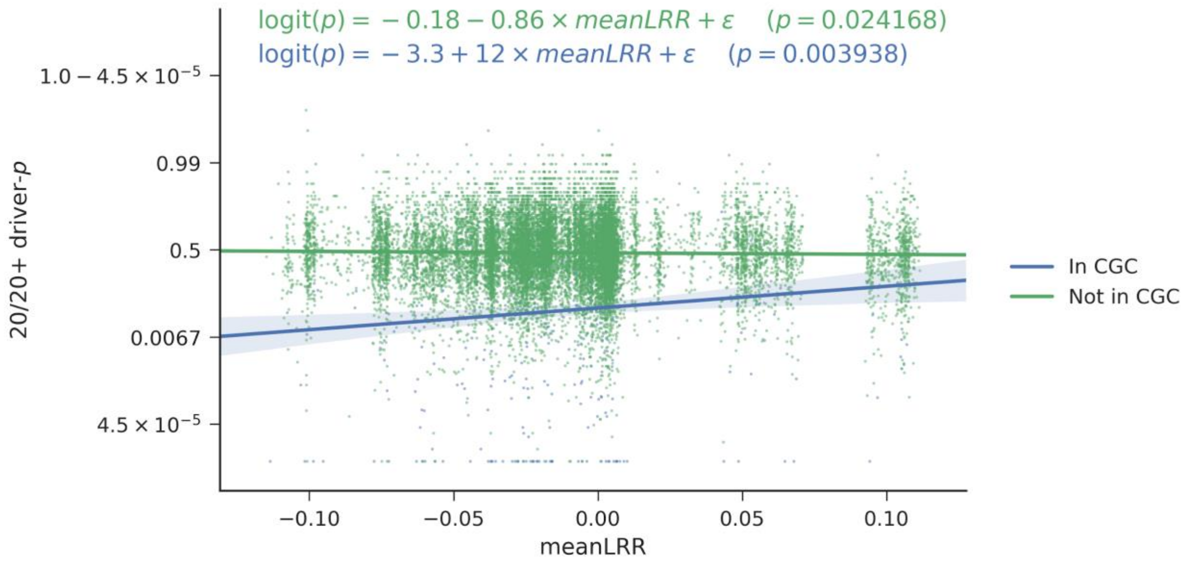

Supplementary Figure 7. Linear dependency of 2020+ logit(driver p) -value on mean LRR of the gene in pancancer dataset. LRR values computed from 1699 samples in this study. Driver p-values are downloaded from Supplementary Table 4 of [1]. Linear least squares model to logit(p) values is fitted separately to genes in/not in COSMIC Cancer Gene Census.

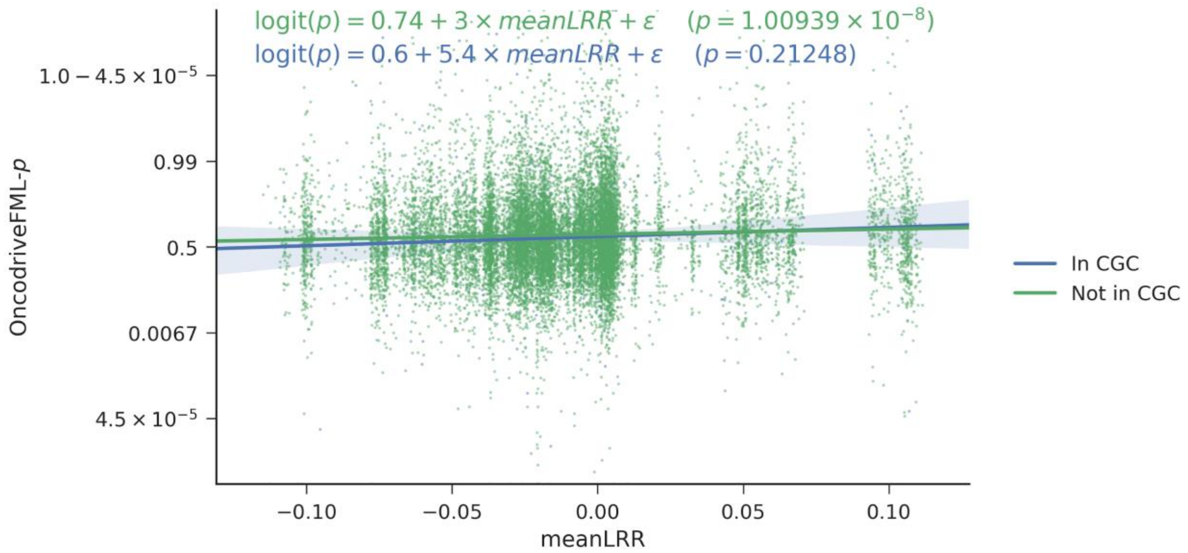

Supplementary Figure 8. Linear dependency of OncodriveFML logit(p) -value on current study WGS data on mean LRR of the gene. LRR values computed from 1699 samples in this study. Linear least squares model to logit(p) values is fitted separately to genes in/not in COSMIC Cancer Gene Census.

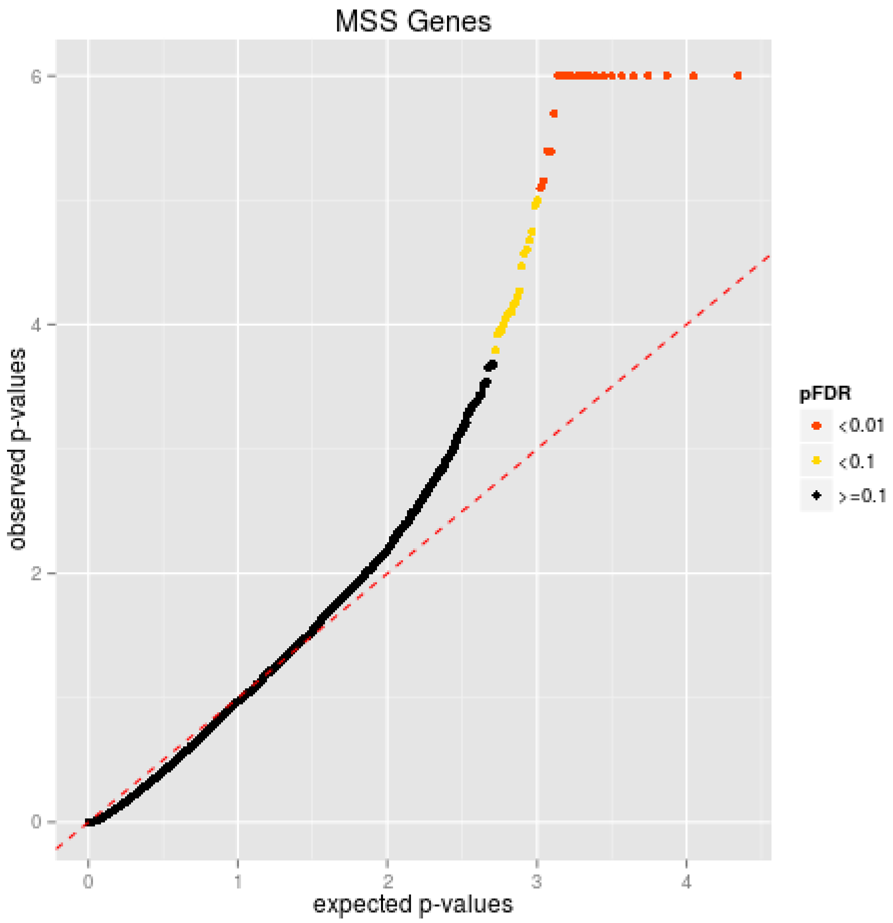

Supplementary Figure 9. QQ-plot showing the -$\log_{10}$(p) values of the OncodriveFML analysis of 234 MSS CRCs plotted against expected -$\log_{10}$(p) values. Adjusted p-values (FDR, Benjamini-Hochberg method) indicated with red (FDR≤1%), yellow (1%<FDR<10%) and black (FDR≥10%).

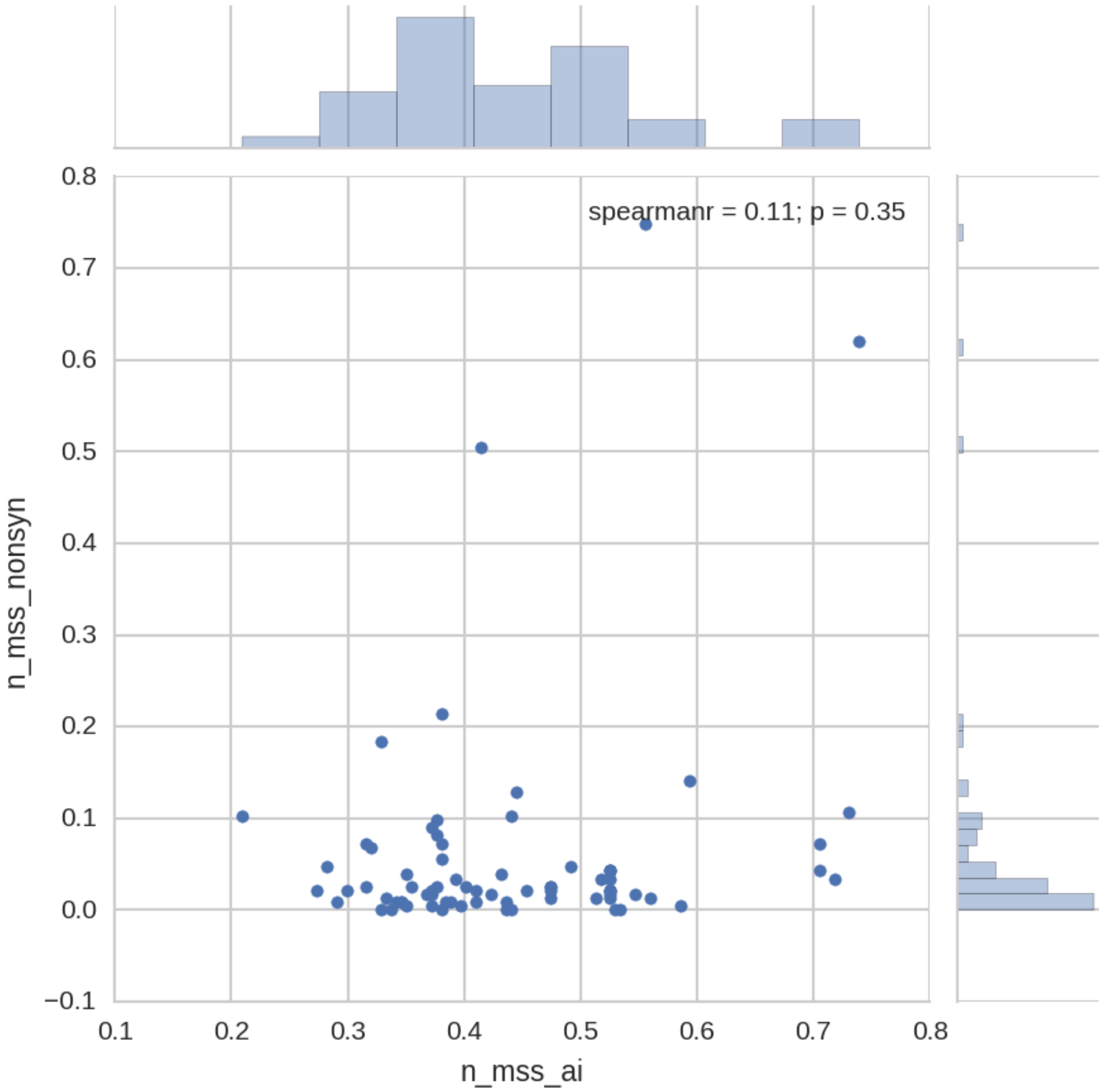

Supplementary Figure 10 Somatic allelic imbalance events and somatic SNVs and indels in the 74 genes (depicted as blue dots) associated with colorectal cancer in COSMIC or identified significant in the OncodriveFML analysis (<10% FDR). Data for the 234 microsatellite stable colorectal cancers shown.

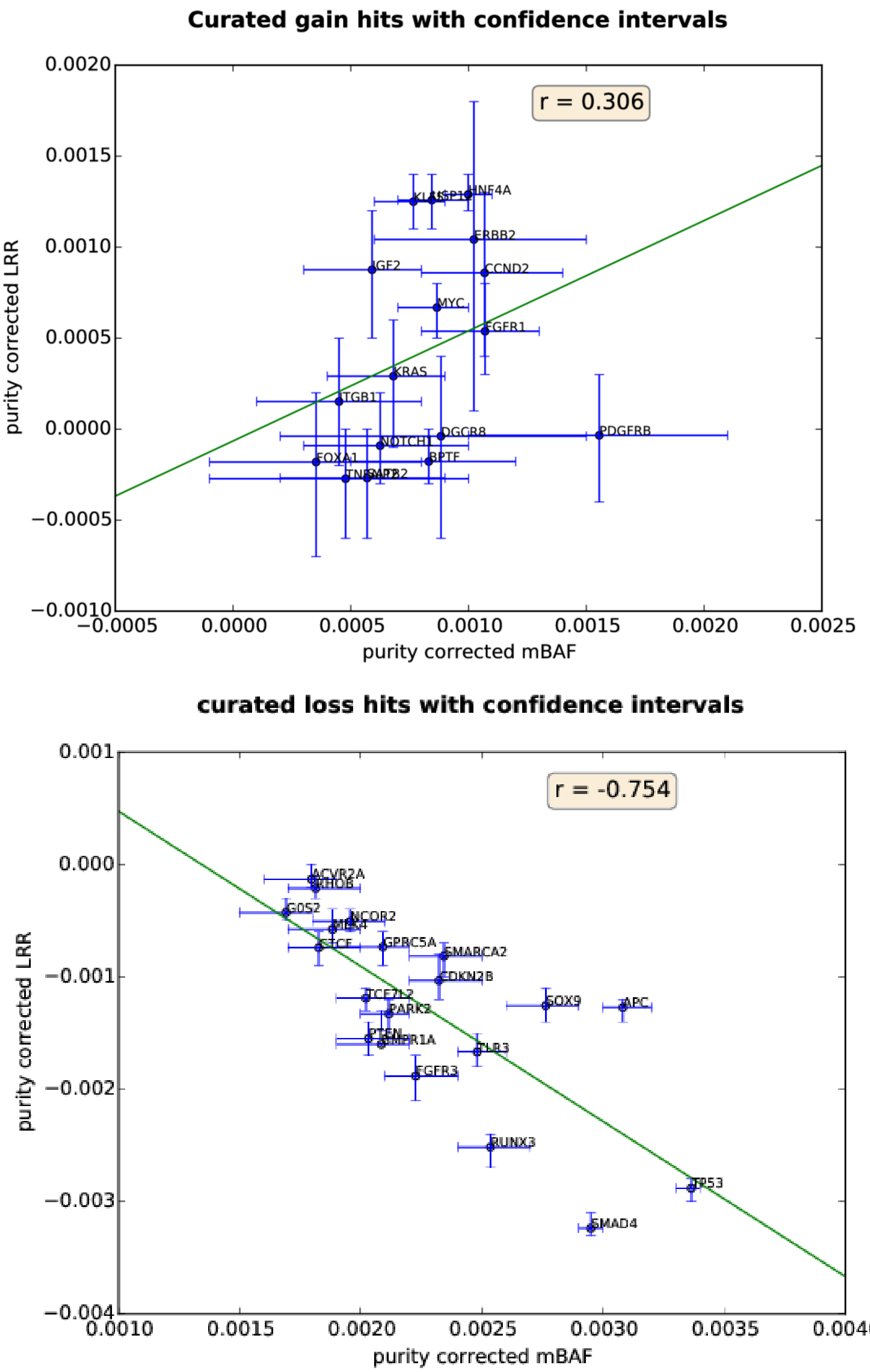

Supplementary Figure 11: Mean B-allele frequency and log-R-ratio for curated gain and loss peaks corrected for tumor purity. Error bars representing 95% confidence intervals were obtained by bootstrapping.

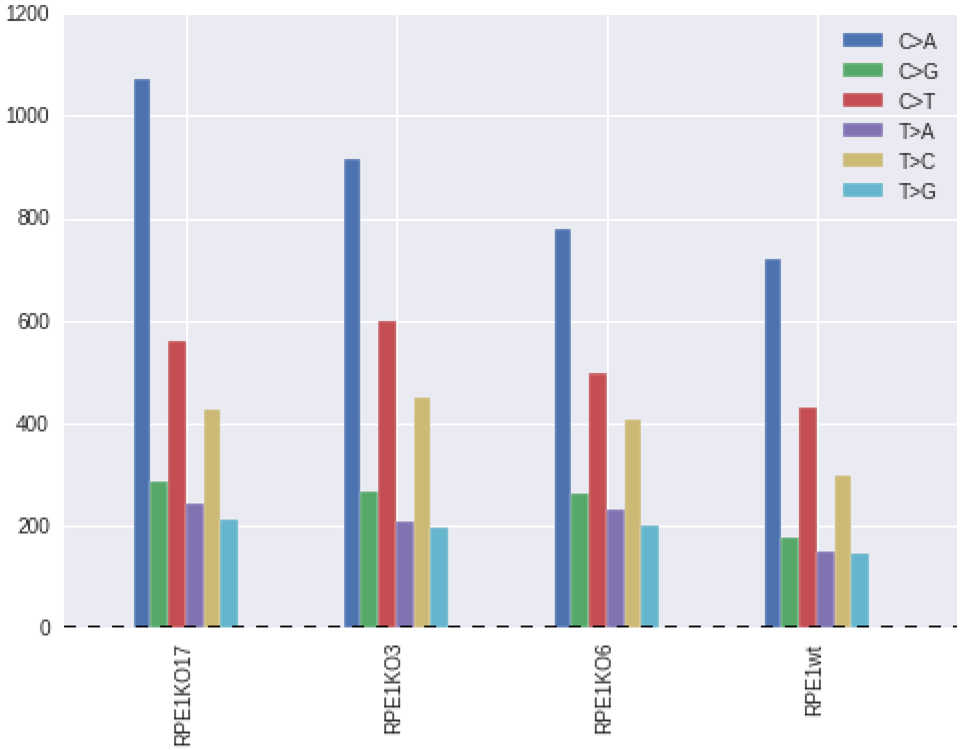

Supplementary Figure 12: Number of single base substitutions in genomes with *TP53*-knockout cell lines separated by substitution type.

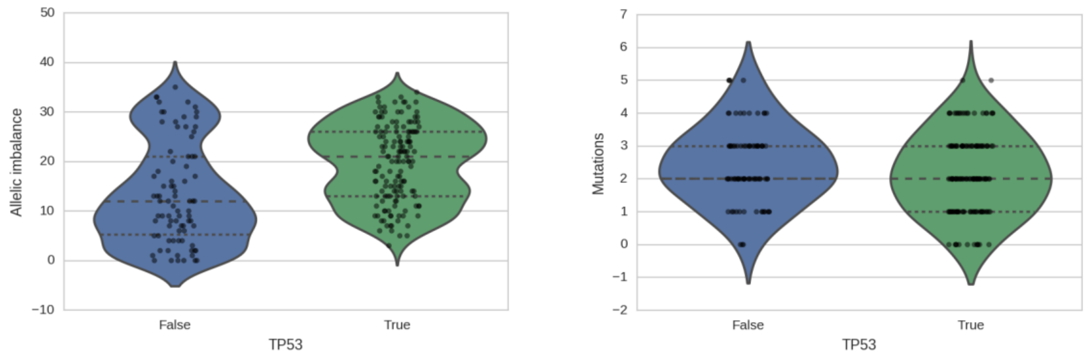

Supplementary Figure 13. Left: allelic imbalance events in the set of 38 genes targeted by AI events in tumors without (blue; n=82) and with a nonsynonymous mutation (green; n=145) in *TP53*. Right: nonsynonymous mutations in the set of 42 significantly mutated genes. *TP53* mutation not counted in the set of *TP53* mutated tumors. Data shown for 227/234 MSS tumors where both WGS and array data were available.

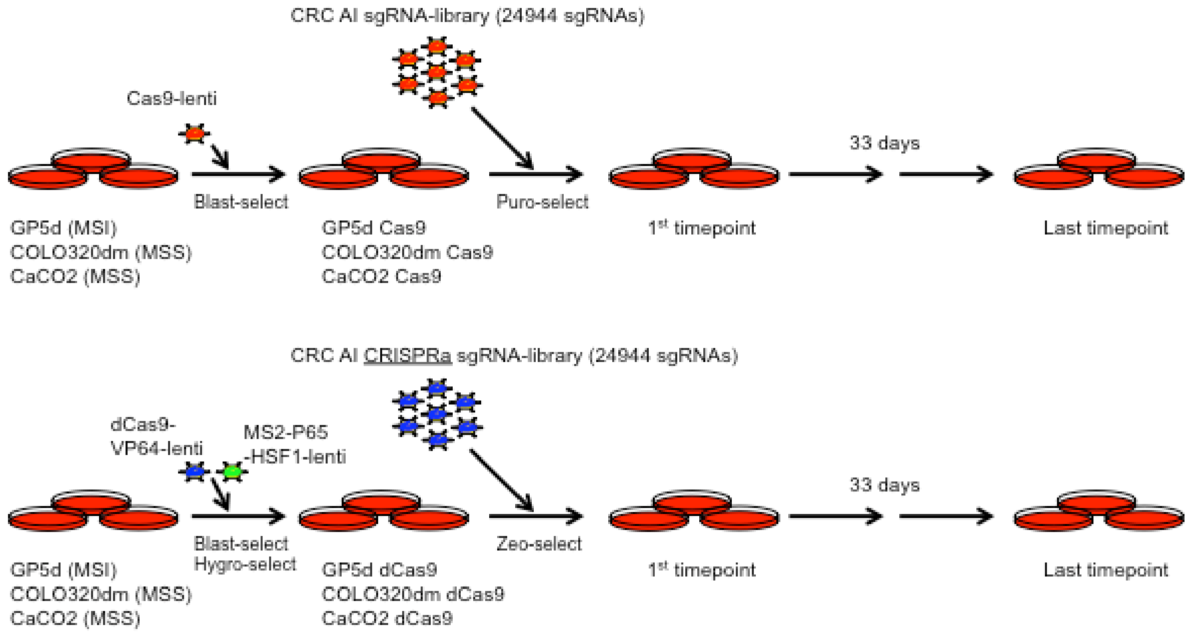

Supplementary Figure 14. Schematic design of the pooled CRISPR knockout and CRISPR activation screens. Both experiments targeted same set of genes but the cutting CRISPRko library targetted protein coding regions and CRISPRa library targeted promoter regions. See methods below for details.

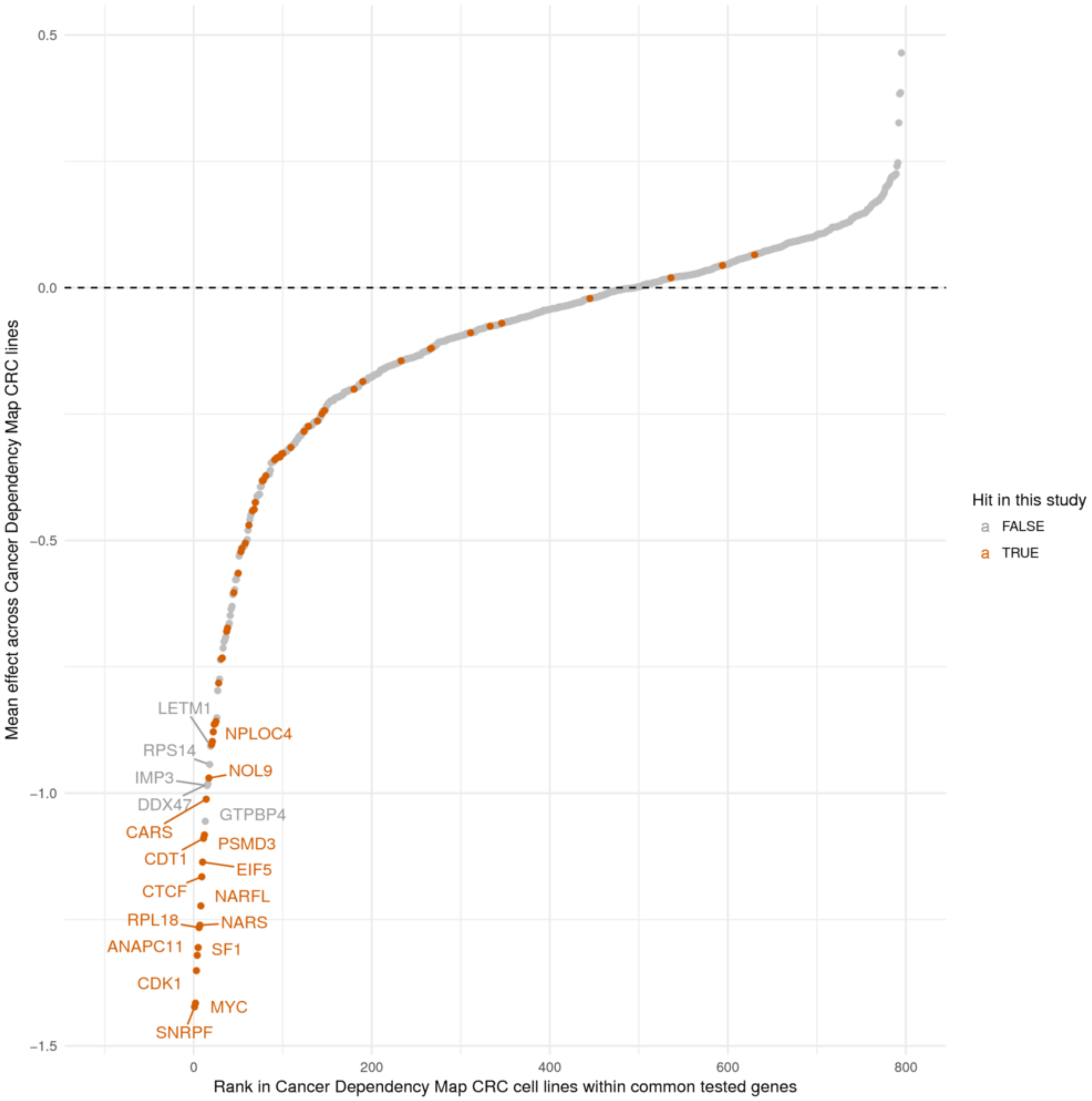

Supplementary Figure 15: Comparison of CRISPR/Cas9 knock-out screens. The mean effect (y-axis) across the Cancer Dependency Map CRC cell lines and its rank (x-axis) are shown for 795 genes in AI peak regions. A point is colored red if the gene was identified as required for cell growth in our screen in at least one cell line. The twenty genes whose knock-out had the largest mean effect among the 795 genes across the CRC lines screened by the Cancer Dependency Map Project are named.

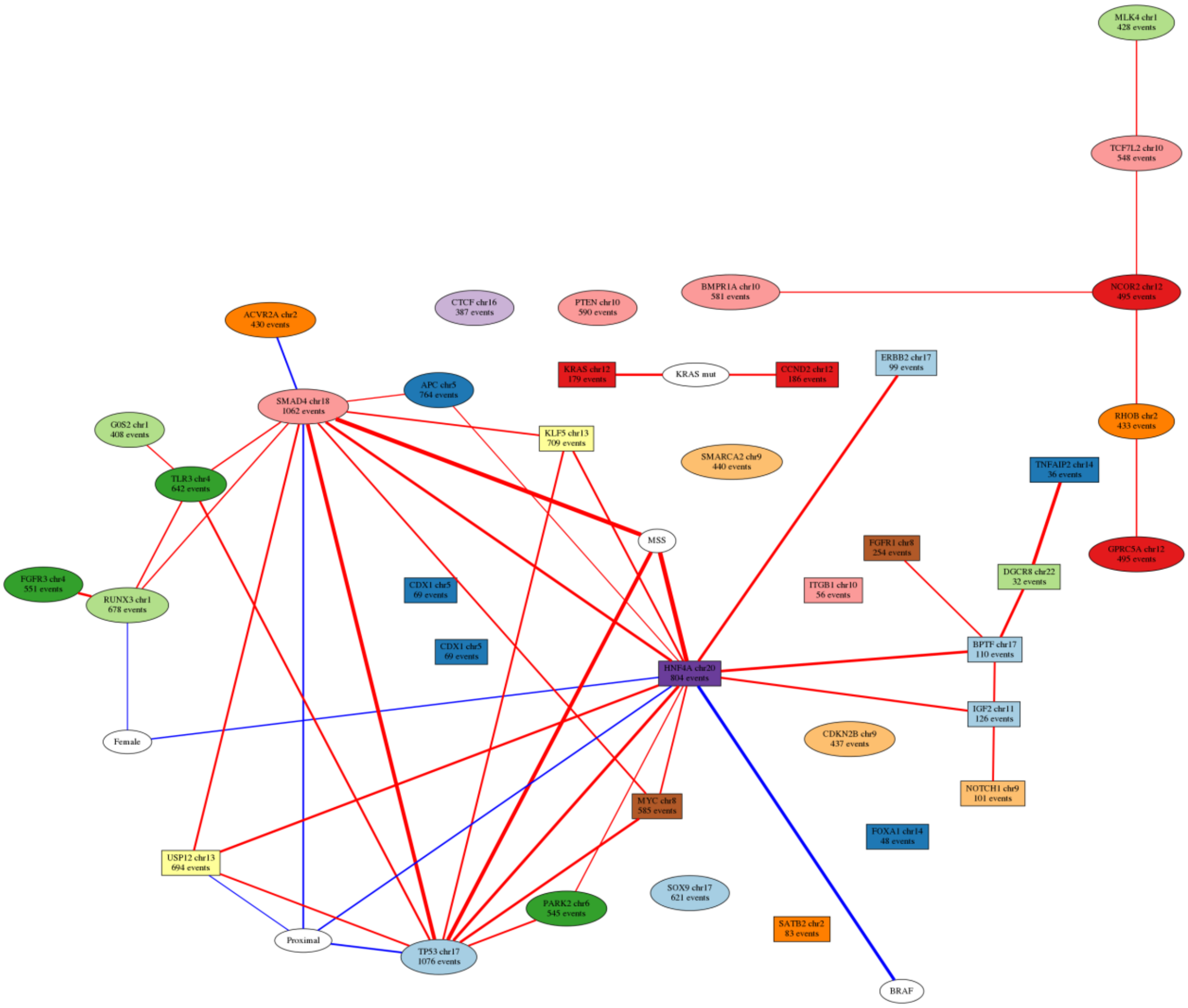

Supplementary Figure 16. Many but not all prominent peaks with candidate target genes associated with each other and clinical phenotypes. Oval and rectangle nodes represent loss and gain peaks, respectively. Nodes are colored according to chromosome (colors cycled). Non-colored nodes represent clinical phenotypes. An edge is drawn between nodes if genome wide corrected association p-value <0.001. For 46 phenotypes considered, the overall family wise Bonferroni corrected p-value is hence <0.05. Width of edge is proportional to effect size. Red and blue edges represent positive and negative effects respectively.

FOXA1

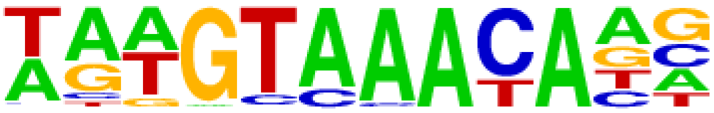

HNF4A

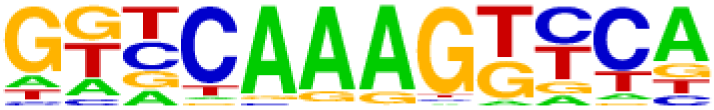

KLF5

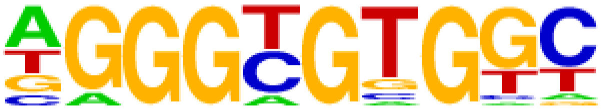

MYC

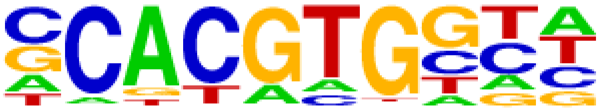

TCF7L2

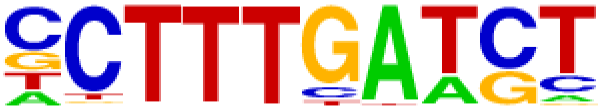

Supplementary Figure 17. Sequence logos of the motifs used in defining transcription factor binding sites from merged PeakXus peak calls.

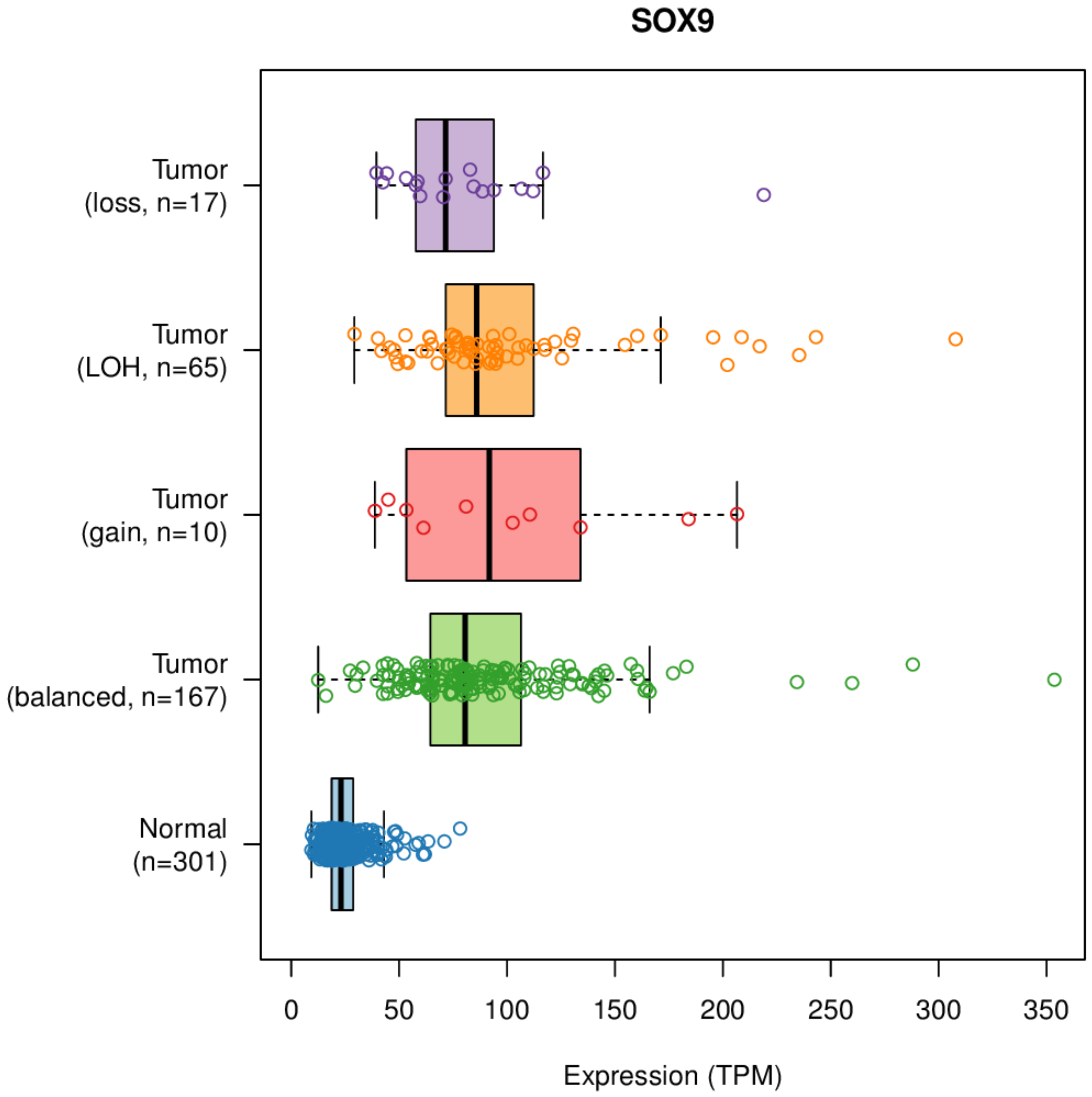

Supplementary Figure 18: Expression level of SOX9 in 301 normal samples and in 259 tumors with different copy genomic copy numbers on the locus.

Supplementary Tables

Supplementary Table 1. Loss events detected in array data at 21 fragile sites (probability being a tumor supressor <15%)[2]. Number of alleles without (no ev) and with (event) focal loss events after correcting for site-spanning AI events reported for MSS and MSI tumors. Odds ratio and Fisher's *p*-value given for MSS/MSI comparison. Preference is either MSI or MSS whenever *p*<0.05.

Gene/Region	Site (GRCh37)	MSS no ev	MSS event	MSI no ev	MSI event	OR	Fisher's p	Preference
DAB1	1:57463578-58716211	2046	11	192	1	1.03	1.00E+00	-
LRP1B	2:140988995-142889270	1825	14	175	1	1.34	1.00E+00	-
FHIT	3:59735035-61237133	1728	227	193	75	**0.34**	**2.94E-11**	**MSI**
TUSC7	3:116428634-116435885	1969	0	268	0	-	1.00E+00	-
4p16	4:53226-88099	1886	0	176	0	-	1.00E+00	-
4p16	4:53276-156490	1838	48	173	3	1.51	7.98E-01	-
CCSER1	4:91048683-92523369	1731	97	150	24	**0.35**	**7.07E-05**	**MSI**
PDE4D	5:58264865-59189621	1939	45	153	4	-	7.79E-01	-
PARK2	6:161768589-163148834	1892	180	225	8	**2.68**	**3.48E-03**	**MSS**
IMMP2L	7:110303109-111202347	1778	10	158	2	0.44	2.58E-01	-
CNTNAP2	7:145813452-148118086	1781	6	158	1	0.53	4.50E-01	-
PTPRD	9:8314245-10612509	1588	15	123	0	-	6.19E-01	-
DLG2	11:83166057-85338314	1669	8	118	2	0.28	1.40E-01	-
IQSEC3	12:186541-280494	1753	29	123	1	2.03	7.18E-01	-
ANKS1B	12:99129070-100378432	1763	5	124	0	-	1.00E+00	-
GPHN	14:66974124-67648525	1479	10	100	5	**0.14**	**1.99E-03**	**MSI**
CHRFAM7A	15:30653442-30685864	1525	0	124	0	-	1.00E+00	-
15q13	15:30395749-32727250	1528	0	124	0	-	1.00E+00	-
16p13	16:6069131-7763340	1600	332	215	40	1.12	5.95E-01	-
WWOX	16:78133550-79246564	1868	65	217	39	**0.19**	**1.99E-12**	**MSI**
MACROD2	20:13976145-16033841	1830	234	124	39	**0.41**	**1.68E-05**	**MSI**

Supplementary Table 2. Association between estimated somatic copy-number and gene expression for the 38 curated genes and the respective AI peak region. Positive Beta (LRR) values suggest that copy-number gains increase expression and losses decrease it.

**Chr**	**Region start**	**Region end**	**Gene**	**ENSG**	**Adjusted P (LRR)**	**Beta (LRR)**	**Adjusted P (tumor%)**	**Beta (tumor%)**
2	147764123	148964451	*ACVR2A*	ENSG00000121989	7.07E-02	1.27	4.92E-01	0.35
5	111930150	112146117	*APC*	ENSG00000134982	1.90E-14	2.43	5.46E-01	0.26
10	88639910	88730177	*BMPR1A*	ENSG00000107779	8.51E-10	1.98	8.51E-01	0.08
17	65508813	66178894	*BPTF*	ENSG00000171634	3.67E-08	1.65	2.87E-01	0.35
12	4274107	4388084	*CCND2*	ENSG00000118971	9.96E-09	3.77	6.48E-01	-0.38
9	22053591	22164991	*CDKN2B*	ENSG00000147883	5.42E-01	0.34	9.52E-07	-2.86
5	149443298	149709644	*CDX1*	ENSG00000113722	4.05E-02	1.46	2.14E-01	0.74
16	67426869	67727069	*CTCF*	ENSG00000102974	4.19E-04	1.34	2.16E-01	0.38
22	20057458	20742450	*DGCR8*	ENSG00000128191	2.22E-07	1.35	7.79E-01	0.11
17	37767453	38131187	*ERBB2*	ENSG00000141736	3.02E-16	2.51	8.54E-03	0.95
8	38277867	38430234	*FGFR1*	ENSG00000077782	2.73E-01	0.42	2.87E-07	-2.31
4	1718116	1797358	*FGFR3*	ENSG00000068078	5.06E-03	1.35	2.17E-01	0.89
14	38064738	39001476	*FOXA1*	ENSG00000129514	4.27E-11	6.15	1.21E-02	2.53
1	209722772	210304319	*G0S2*	ENSG00000123689	3.04E-01	1.21	1.43E-03	-2.81
12	12932932	13070752	*GPRC5A*	ENSG00000013588	3.03E-04	2.38	3.57E-01	0.57
20	43048685	43223862	*HNF4A*	ENSG00000101076	5.59E-65	2.77	3.72E-01	0.32
11	2167981	2232229	*IGF2*	ENSG00000167244	5.63E-05	5.57	3.04E-01	-2.17
10	33135952	34237166	*ITGB1*	ENSG00000150093	4.37E-09	2.12	1.81E-01	-0.40
13	73619229	73684920	*KLF5*	ENSG00000102554	1.76E-02	0.57	1.20E-02	0.84
12	25304707	25518438	*KRAS*	ENSG00000133703	4.80E-11	2.23	7.37E-01	0.13
1	233292257	233561654	*MLK4*	ENSG00000143674	3.87E-14	2.39	2.16E-01	0.38
8	128409511	128571059	*MYC*	ENSG00000136997	5.04E-09	1.98	5.91E-01	0.30
12	124855274	124907914	*NCOR2*	ENSG00000196498	1.01E-06	1.57	6.22E-01	0.17
9	139387618	139616742	*NOTCH1*	ENSG00000148400	8.87E-05	0.77	6.14E-01	-0.20
6	162832336	162853242	*PARK2*	ENSG00000185345	6.46E-01	-0.23	4.42E-01	0.60
5	149443298	149709644	*PDGFRB*	ENSG00000113721	1.82E-01	-1.06	1.50E-08	-2.94
10	89507111	90105898	*PTEN*	ENSG00000171862	8.94E-31	2.61	5.21E-01	0.24
2	20588862	20716754	*RHOB*	ENSG00000143878	8.55E-08	3.95	8.25E-01	-0.15
1	25069939	25298408	*RUNX3*	ENSG00000020633	9.79E-01	0.01	4.61E-01	-0.58
2	199469815	200160277	*SATB2*	ENSG00000119042	1.64E-03	2.80	9.96E-01	-0.01
18	48363299	48981991	*SMAD4*	ENSG00000141646	1.42E-36	2.10	3.94E-01	0.29
9	1117444	1501977	*SMARCA2*	ENSG00000080503	1.55E-16	2.26	9.87E-01	0.01
17	70425875	70475574	*SOX9*	ENSG00000125398	1.47E-02	0.89	1.16E-03	1.21
10	114742835	114840745	*TCF7L2*	ENSG00000148737	8.84E-07	1.72	9.78E-03	0.74
4	186887218	187116227	*TLR3*	ENSG00000164342	1.49E-20	4.27	9.87E-01	0.02
14	103449773	104029819	*TNFAIP2*	ENSG00000185215	3.10E-01	0.44	5.23E-03	-1.53
17	7607019	7721542	*TP53*	ENSG00000141510	1.94E-08	1.93	4.10E-02	1.04
13	27497956	27563038	*USP12*	ENSG00000152484	4.71E-31	1.94	8.51E-01	0.08

Supplementary Table 3. Significant genes (FDR<0.1) in OncodriveFML analysis of 234 MSS tumors. Ensembl gene id (ENSG), total number of SNVs, number of unique SNVs per position and base change, number of mutated tumors, OncodriveFML *p*-value and *q*-value adjusted for multiple testing with the Benjamini-Hochberg method reported for each gene.

**Gene**	**ENSG**	**SNVs**	**Unique SNVs**	**Tumors**	**p**	**q**
BRAF	ENSG00000157764	132	117	90	1.00E-06	0.00138425
KRAS	ENSG00000133703	153	42	135	1.00E-06	0.00138425
CTNNB1	ENSG00000168036	30	30	26	1.00E-06	0.00138425
PIK3CA	ENSG00000121879	94	64	79	1.00E-06	0.00138425
ARID1A	ENSG00000117713	51	51	43	1.00E-06	0.00138425
NRAS	ENSG00000213281	59	48	28	1.00E-06	0.00138425
AMER1	ENSG00000184675	38	32	32	1.00E-06	0.00138425
TP53	ENSG00000141510	171	92	150	1.00E-06	0.00138425
PCDHA1	ENSG00000204970	385	382	184	1.00E-06	0.00138425
APC	ENSG00000134982	239	148	158	1.00E-06	0.00138425
PCDHA3	ENSG00000255408	346	343	176	1.00E-06	0.00138425
PCDHA2	ENSG00000204969	362	359	179	1.00E-06	0.00138425
PCDHA4	ENSG00000204967	324	321	169	1.00E-06	0.00138425
FBXW7	ENSG00000109670	143	135	94	1.00E-06	0.00138425
SMAD2	ENSG00000175387	37	37	35	1.00E-06	0.00138425
SMAD4	ENSG00000141646	50	40	45	1.00E-06	0.00138425
OBSCN	ENSG00000154358	192	192	127	2.00E-06	0.002605647059
MLK4	ENSG00000143674	38	38	37	4.00E-06	0.004662736842
SOX9	ENSG00000125398	16	16	15	4.00E-06	0.004662736842
ARSI	ENSG00000183876	6	6	5	7.00E-06	0.0077518
ATM	ENSG00000149311	98	98	73	8.00E-06	0.008437333333
PCDHGA1	ENSG00000204956	284	280	143	1.00E-05	0.01006727273
PCBP1	ENSG00000169564	13	2	13	1.10E-05	0.01059252174
PCDHGA2	ENSG00000081853	277	273	140	1.80E-05	0.016611
BCL9L	ENSG00000186174	14	13	13	2.10E-05	0.01860432
MAP2K4	ENSG00000065559	59	58	48	2.50E-05	0.02129615385
LOXHD1	ENSG00000167210	166	163	116	2.70E-05	0.022148
ARMC12	ENSG00000157343	6	6	6	3.40E-05	0.026894
MAD2L1	ENSG00000164109	6	6	6	5.40E-05	0.04124110345
GNAS	ENSG00000087460	97	93	72	6.00E-05	0.044296

DUSP26	ENSG00000133878	12	11	10	6.60E-05	0.04715380645
PDX1	ENSG00000139515	4	4	4	7.00E-05	0.04844875
CNGA4	ENSG00000132259	8	8	8	8.00E-05	0.05211294118
AAR2	ENSG00000131043	17	17	11	8.00E-05	0.05211294118
MMP27	ENSG00000137675	10	10	10	8.40E-05	0.0531552
BRD7	ENSG00000166164	24	23	23	9.00E-05	0.05537
PCDHA5	ENSG00000204965	299	297	163	0.0001	0.05985945946
ERBB2	ENSG00000141736	21	18	20	0.00011	0.06246871795
SBK1	ENSG00000188322	37	37	37	0.00011	0.06246871795
PRKAG1	ENSG00000181929	12	11	12	0.00012	0.06482341463
KLF5	ENSG00000102554	27	26	22	0.00012	0.06482341463
CHUK	ENSG00000213341	33	33	30	0.00016	0.08437333333

Supplementary Table 4. Nonsynonymous and truncating somatic mutations of 234 MSS CRCs in the 42 genes associated with colorectal cancer in the COSMIC cancer gene census.

Gene	Nonsynonymous	Nonsyn %	Truncs	Truncs %
AKT1	2	1%	0	0%
APC	179	76%	179	76%
AXIN1	2	1%	2	1%
AXIN2	5	2%	3	1%
BRAF	25	11%	0	0%
C2orf44	2	1%	1	0%
CSF3R	3	1%	2	1%
CTNNB1	9	4%	0	0%
CUX1	2	1%	1	0%
EIF3E	0	0%	0	0%
EP300	5	2%	2	1%
ERBB3	13	6%	5	2%
FBXW7	30	13%	8	3%
GRIN2A	11	5%	4	2%
HIF1A	0	0%	0	0%
KRAS	121	52%	0	0%
MAP2K1	3	1%	0	0%
MAP2K4	8	3%	3	1%
MAX	0	0%	0	0%
MDM2	2	1%	1	0%
MLH1	2	1%	1	0%
MSH2	2	1%	2	1%
MSH6	1	0%	0	0%
MUTYH	0	0%	0	0%
PIK3CA	43	18%	1	0%
PIK3R1	6	3%	1	0%
PMS1	4	2%	3	1%
PMS2	2	1%	0	0%
POLE	6	3%	5	2%
PTPRK	6	3%	2	1%
RAD21	4	2%	0	0%
RSPO2	0	0%	0	0%
RSPO3	1	0%	0	0%
SMAD2	11	5%	6	3%
SMAD3	6	3%	2	1%

SMAD4	27	12%	7	3%
TBL1XR1	0	0%	0	0%
TCF7L2	21	9%	14	6%
TGFBR2	6	3%	5	2%
TP53	148	63%	32	14%
UBR5	8	3%	3	1%
VTI1A	1	0%	1	0%

Supplementary Table 5: Number of single base substitutions in genomes with TP53-knockout cell lines separated by substitution type.

	C>A	C>G	C>T	T>A	T>C	T>G
RPE1KO17	1072	285	560	242	425	213
RPE1KO3	915	266	598	208	450	198
RPE1KO6	777	263	498	230	407	199
RPE1wt	722	177	429	149	297	144

**TF name/ cell line**			
KLF5/ GP5D	**Number of peaks:** 855, **Read count:** 52479079, **UMI count:** 12755580<br>**Antibody:** sc-22797x		
KLF5/ GP5D	**1st MEME motif**<br>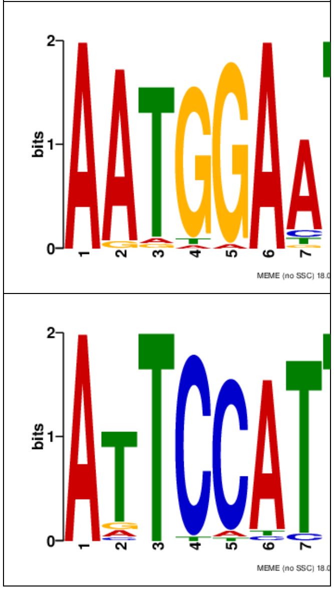<br>E-value = 1.6e-341<br>sites = 336/1000	**2nd MEME motif**<br>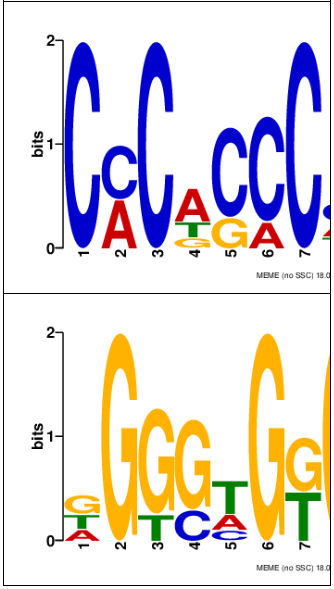<br>E-value = 1.3e-336<br>sites = 913/1000	**3rd MEME motif**<br>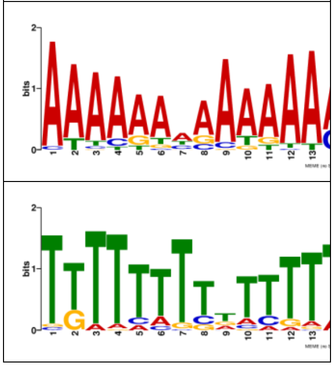<br>E-value = 1.5e-063<br>sites = 56/1000
HNF4A/ GP5D	**Number of peaks:** 43447, **Read count:** 78879518, **UMI count:** 27262445<br>**Antibody:** sc-8987x		
HNF4A/ GP5D	**1st MEME motif**<br>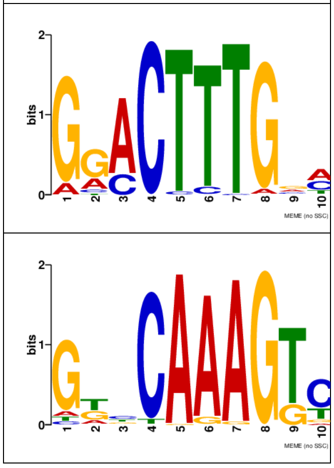<br>E-value = 1.2e-1232<br>sites = 906/1000	**2nd MEME motif**<br>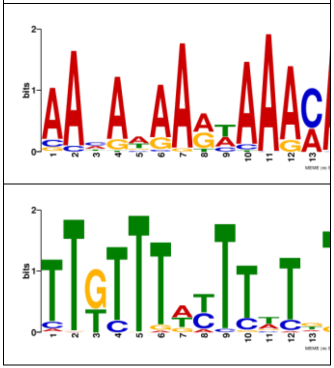<br>E-value = 1.7e-110<br>sites = 111/1000	**3rd MEME motif**<br>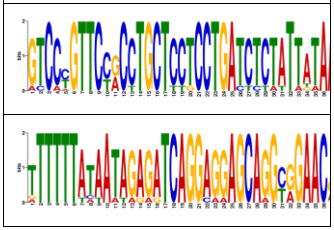<br>E-value = 1.7e-090<br>sites = 10/1000
FOXA1/ GP5D	**Number of peaks:** 178533, **Read count:** 80669121, **UMI count:** 38435746<br>**Antibody:** ab23738		

FOXA1/<br>GP5D	**1st MEME motif**<br>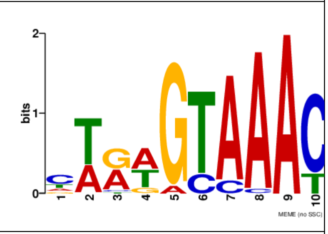<br>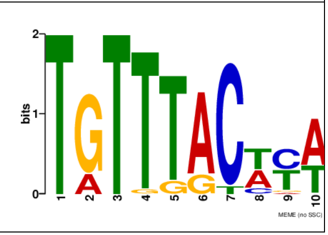<br>E-value = 6.0e-1190<br>sites = 1000/1000	**2nd MEME motif**<br>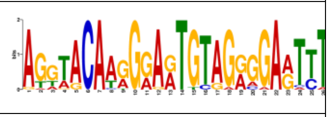<br>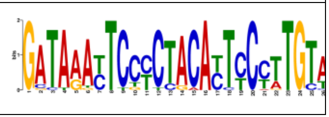<br>E-value = 2.1e-121<br>sites = 22/1000	**3rd MEME motif**<br>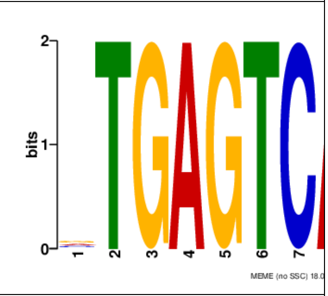<br>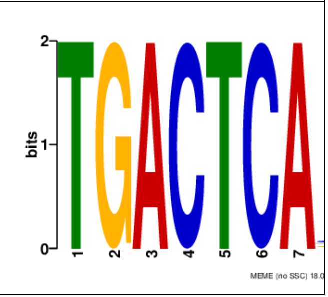<br>E-value = 2.8e-064<br>sites = 131/1000
MYC/<br>GP5D	**Number of peaks:** 63855, **Read count:** 78310947, **UMI count:** 31488097<br>**Antibody:** Millipore, 06-340		
MYC/<br>GP5D	**1st MEME motif**<br>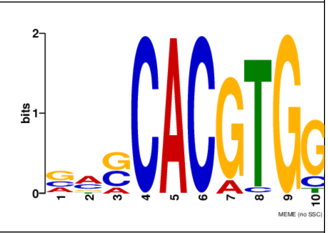<br>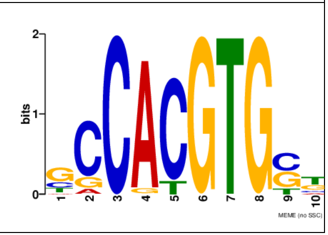<br>E-value = 3.2e-279<br>sites = 467/1000	**2nd MEME motif**<br>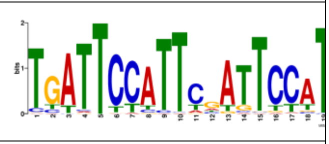<br>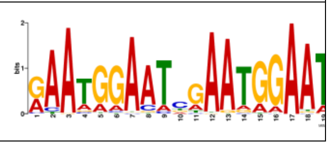<br>E-value = 9.2e-341<br>sites = 76/1000	**3rd MEME motif**<br>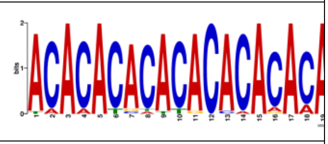<br>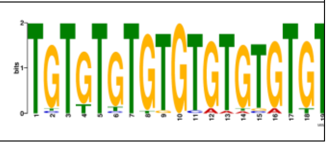<br>E-value = 5.3e-147<br>sites = 26/1000
TCF7L2/GP<br>5D	**Number of peaks:** 13312, **Read count**: 39781128, **UMI count**: 7781298<br>**Antibody:** Xalpha biologicals, X1070M		
TCF7L2/GP<br>5D	**1st MEME motif**<br>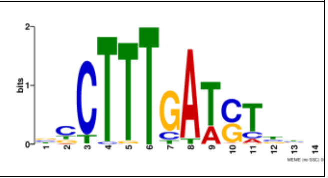	**2nd MEME motif**<br>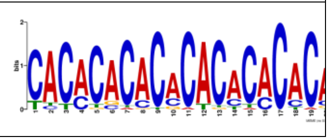	**3rd MEME motif**<br>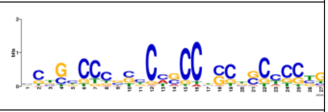<br>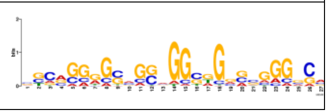

	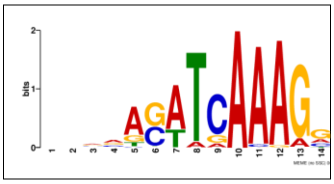<br>E-value = 9.8e-896<br>sites = 916/1000	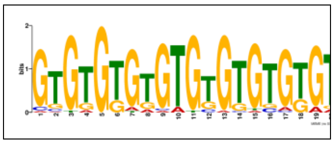<br>E-value = 1.1e-113<br>sites = 33/1000	E-value = 4.6e-088<br>sites = 114/1000

Supplementary Table 6: Summary of the GP5D ChIP-Nexus experiments. Three of the best MEME-motifs found from the set of top 1000 peaks reported by PeakXus are shown. MEME was evoked with parameters "-dna -mod anr -nmotifs 5 -maxsites 1000 -minw 4 -maxw 50 -revcomp".

<table>
<tr><th>TF name/ cell line</th><th colspan="3"></th></tr>
<tr><td>KLF5/ LoVo</td><td colspan="3">Number of peaks: 5129, Read count: 64643770, UMI count: 17864865
Antibody: sc-22797x</td></tr>
<tr><td>KLF5/ LoVo</td><td>1st MEME motif
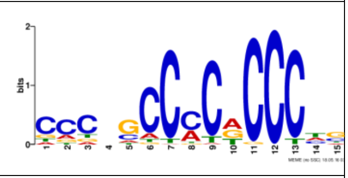
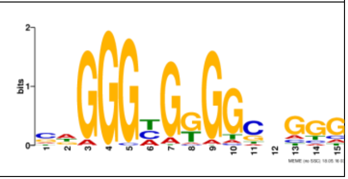
E-value = 8.4e-566
sites = 994/1000</td><td>2nd MEME motif
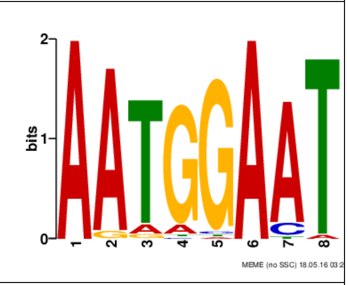
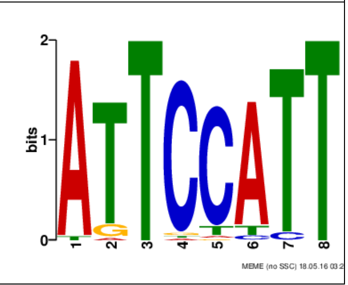
E-value = 2.8e-230
sites = 242/1000</td><td>3rd MEME motif
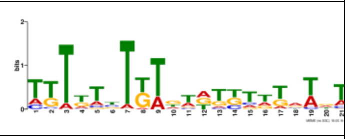
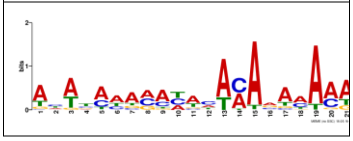
E-value = 1.1e-108
sites = 157/1000</td></tr>
<tr><td>HNF4A/ LoVo</td><td colspan="3">Number of peaks: 53894, Read count: 76565870, UMI count: 26188319
Antibody: sc-8987x</td></tr>
<tr><td>HNF4A/ LoVo</td><td>1st MEME motif
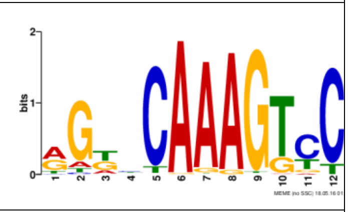
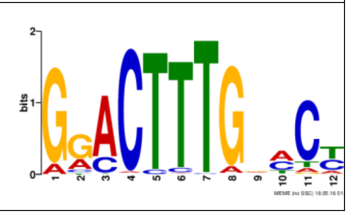
E-value = 3.4e-1257
sites = 974/1000</td><td>2nd MEME motif
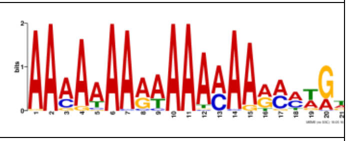
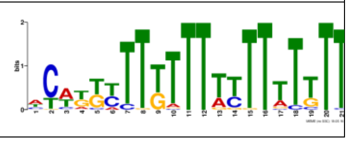
E-value = 1.7e-110
sites = 45/1000</td><td>3rd MEME motif
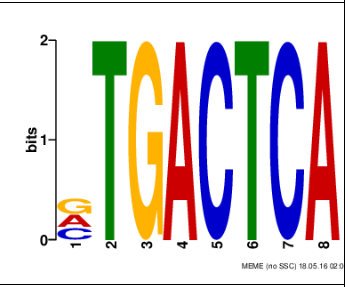
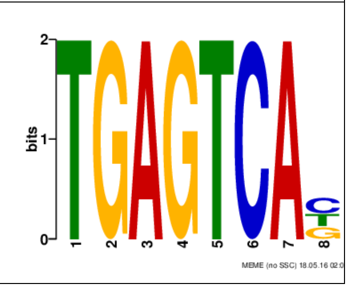
E-value = 1.4e-049
sites = 105/1000</td></tr>
</table>

FOXA1/ LoVo	**Number of peaks:** 183899, **Read count:** 112951859, **UMI count:** 49175367<br>**Antibody:** ab23738		
FOXA1/ LoVo	**1st MEME motif**<br>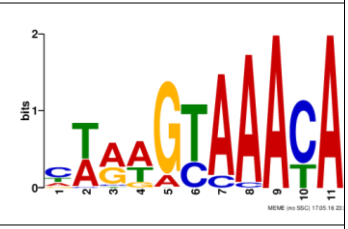<br>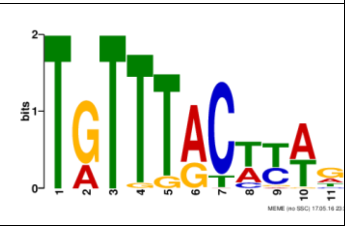<br>E-value = 5.5e-904<br>sites = 999/1000	**2nd MEME motif**<br>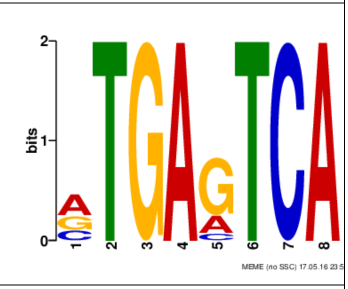<br>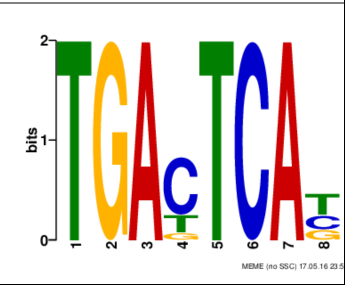<br>E-value = 1.0e-112<br>sites = 225/1000	**3rd MEME motif**<br>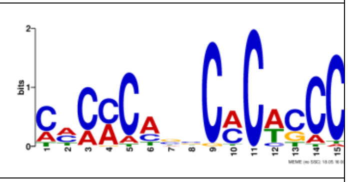<br>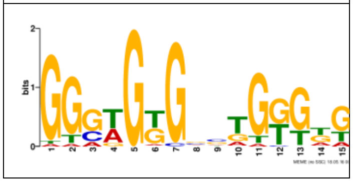<br>E-value = 7.3e-038<br>sites = 88/1000
MYC/ LoVo	**Number of peaks:** 48811, **Read count:** 60330181, **UMI count:** 24112006<br>**Antibody:** Millipore, 06-340		
MYC/ LoVo	**1st MEME motif**<br>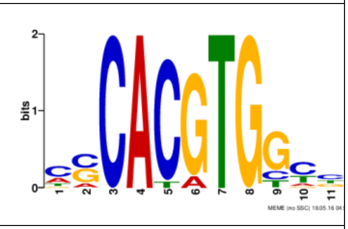<br>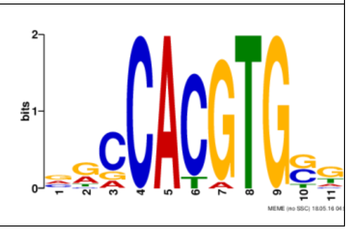<br>E-value = 1.6e-328<br>sites = 527/1000	**2nd MEME motif**<br>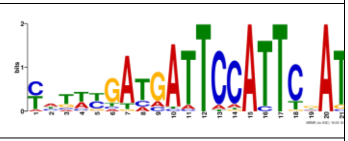<br>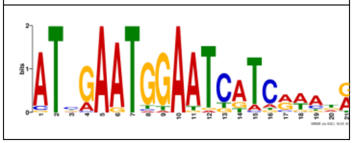<br>E-value = 4.4e-072<br>sites = 27/1000	**3rd MEME motif**<br>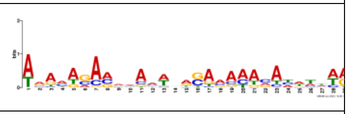<br>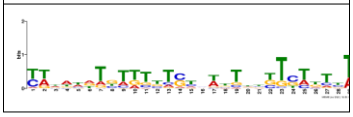<br>E-value = 7.4e-047<br>sites = 77/1000
TCF7L2 / LoVo	**Number of peaks:** 85277, **Read count:** 88388698, **UMI count:** 35176673<br>**Antibody:** Xalpha biologicals, X1070M		
TCF7L2 / LoVo	**1st MEME motif**	**2nd MEME motif**	**3rd MEME motif**

	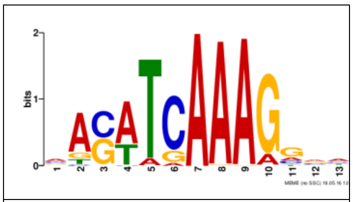<br>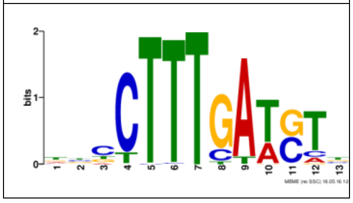<br>E-value = 2.3e-747<br>sites = 798/1000	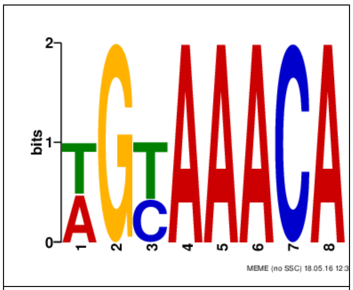<br>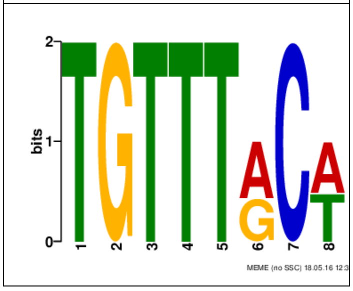<br>E-value = 7.4e-034<br>sites = 128/1000	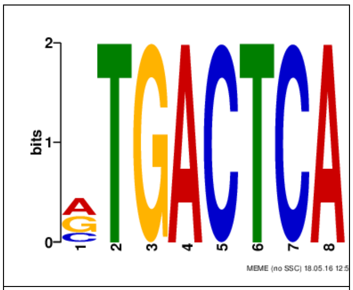<br>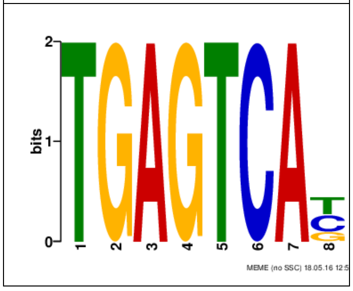<br>E-value = 1.1e-021<br>sites = 76

Supplementary Table 7: Summary of the LoVo ChIP-Nexus experiments. Three of the best MEME-motifs found from the set of top 1000 peaks reported by PeakXus are shown. MEME was evoked with parameters "-dna -mod anr -nmotifs 5 -maxsites 1000 -minw 4 -maxw 50 -revcomp".

**TF name/ cell line**	
FOXA1/ COLO320	**Number of peaks:** 109096, **Read count:** 53799025, **UMI count:** 44232125<br>**Antibody:** ab23738
FOXA1/ COLO320	**1st MEME motif**<br>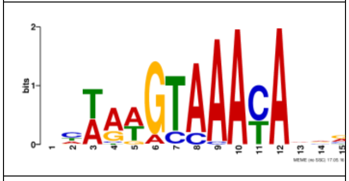<br>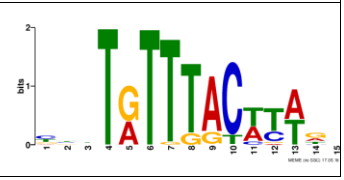<br>E-value = 1.1e-551<br>sites = 728/1000<br><br>**2nd MEME motif**<br>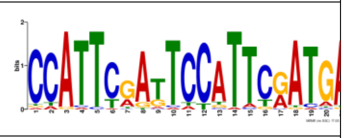<br>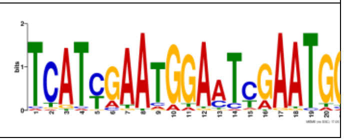<br>E-value = 9.2e-082<br>sites = 31/1000<br><br>**3rd MEME motif**<br>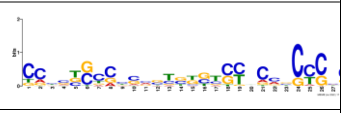<br>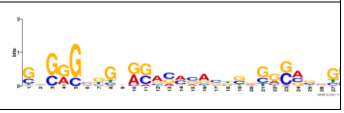<br>E-value = 2.2e-033<br>sites = 59/1000
MYC/ COLO320	**Number of peaks:** 119411, **Read count:** 47593662, **UMI count:** 43101223<br>**Antibody:** Millipore, 06-340
MYC/ COLO320	**1st MEME motif**<br>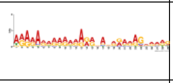<br>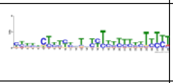<br>E-value = 1.3e-209<br>sites = 226<br><br>**2nd MEME motif**<br>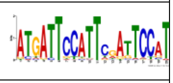<br>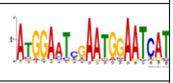<br>E-value = 4.0e-142<br>sites = 47<br><br>**3rd MEME motif**<br>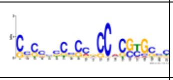<br>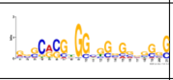<br>E-value = 4.9e-139<br>sites = 256<br><br>**4th MEME motif**<br>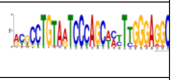<br>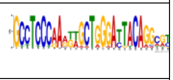<br>E-value = 2.3e-069<br>sites = 14<br><br>**5th MEME motif**<br>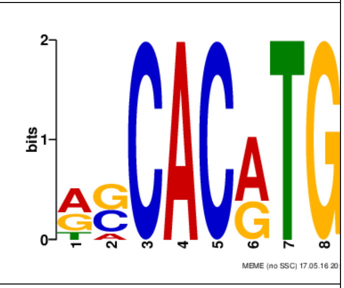<br>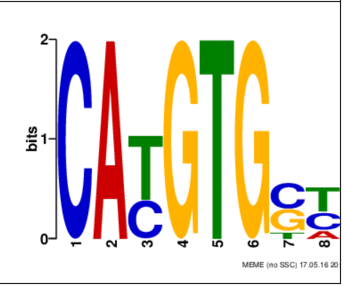<br>E-value = 1.1e-038<br>sites = 213
TCF7L2/ COLO32	**Number of peaks:** 87486, **Read count:** 28465158, **UMI count:** 25207031<br>**Antibody:** Xalpha biologicals, X1070M

0			
TCF7L2/ COLO320	**1st MEME motif**<br>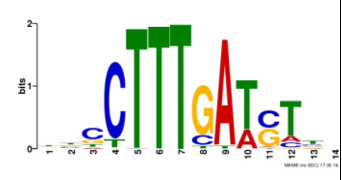<br>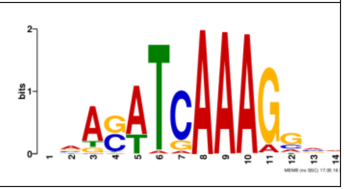<br>E-value = 9.6e-845<br>sites = 843/1000	**2nd MEME motif**<br>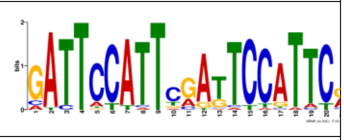<br>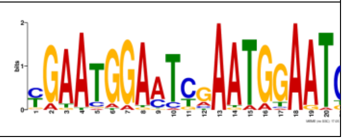<br>E-value = 2.0e-071<br>sites = 28/1000	**3rd MEME motif**<br>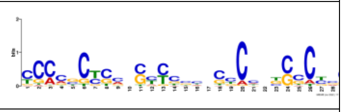<br>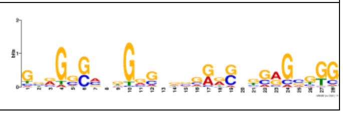<br>E-value = 2.9e-044<br>sites = 62

Supplementary Table 8: Summary of the COLO320 ChIP-Nexus experiments. Three of the best MEME-motifs found from the set of top 1000 peaks reported by PeakXus are shown. MEME was evoked with parameters "-dna -mod anr -nmotifs 5 -maxsites 1000 -minw 4 -maxw 50 -revcomp".

**TF name/ cell line/**			
KLF5/ LoVo	**Number of peaks:** 109040, **Read count:** 51380327, **UMI count:** 39837975<br>**Antibody:** Santa Cruz Biotechnology, sc-22797X		
KLF5/ LoVo	**1st MEME motif**<br>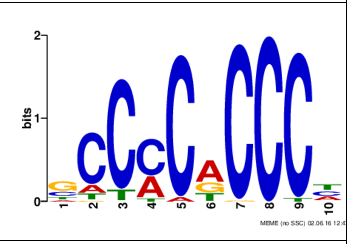<br>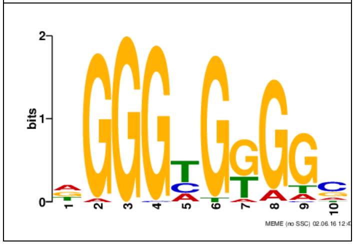<br>E-value = 3.7e-493<br>sites = 999/1000	**2nd MEME motif**<br>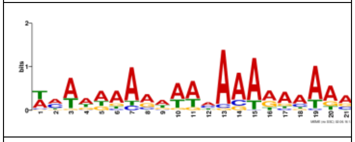<br>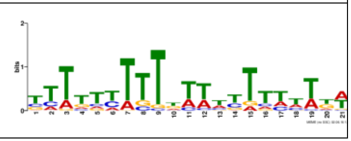<br>E-value = 4.8e-105<br>sites = 176/1000	**3rd MEME motif**<br>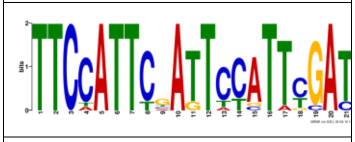<br>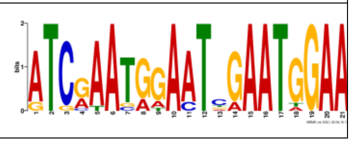<br>E-value = 4.0e-038<br>sites = 13/1000
HNF4A / LoVo	**Number of peaks:** 140480, **Read count:** 27730653, **UMI count:** not used!<br>**Antibody:** Santa Cruz Biotechnology, sc-8987X		
HNF4A / LoVo	**1st MEME motif**<br>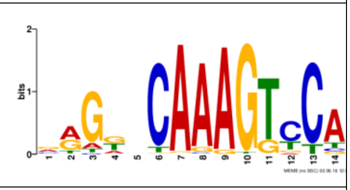<br>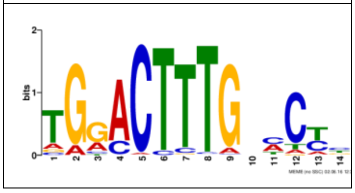<br>E-value = 3.3e-1096<br>sites = 799/1000	**2nd MEME motif**<br>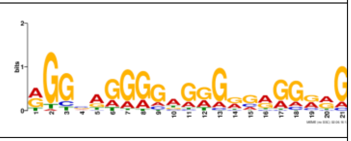<br>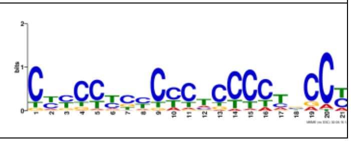<br>E-value = 4.8e-078<br>sites = 187/1000	**3rd MEME motif**<br>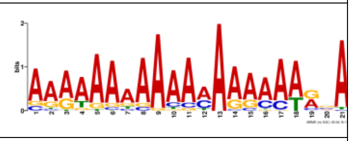<br>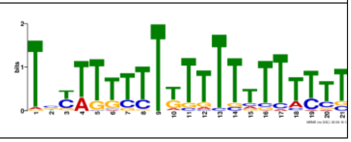<br>E-value = 1.8e-056<br>sites = 51/1000

Supplementary Table 9: Summary of the LoVo ChIP-exo experiments. Three of the best MEME-motifs found from the set of top 1000 peaks reported by PeakXus are shown. MEME was evoked with parameters "-dna -mod anr -nmotifs 5 -maxsites 1000 -minw 4 -maxw 50 -revcomp".

Supplementary Table 10. Association between transcription factors' expression. Based on RNA-seq data from 259 primary colorectal tumors and sleuth Wald tests.

Response (ENSG)	Response	Predictor	P	Adj. P	Beta	SE
ENSG00000129514	FOXA1	HNF4A_expression	3.32E-05	5.36E-04	-0.678	0.163
ENSG00000129514	FOXA1	TCF7L2_expression	9.99E-04	1.03E-02	0.708	0.215
ENSG00000129514	FOXA1	KLF5_expression	1.73E-01	3.86E-01	0.257	0.189
ENSG00000129514	FOXA1	MYC_expression	9.25E-01	9.73E-01	0.014	0.149
ENSG00000129514	FOXA1	FOXA1_LRR	8.15E-06	3.40E-04	4.036	0.905
ENSG00000129514	FOXA1	tumor%	5.44E-02	2.18E-01	1.483	0.771
ENSG00000101076	HNF4A	FOXA1_expression	7.01E-01	9.46E-01	-0.007	0.019
ENSG00000101076	HNF4A	TCF7L2_expression	2.30E-01	5.08E-01	0.079	0.065
ENSG00000101076	HNF4A	KLF5_expression	7.59E-11	8.43E-08	0.341	0.052
ENSG00000101076	HNF4A	MYC_expression	6.50E-05	1.39E-03	0.171	0.043
ENSG00000101076	HNF4A	HNF4A_LRR	2.41E-39	7.14E-36	2.531	0.193
ENSG00000101076	HNF4A	tumor%	9.31E-01	9.75E-01	-0.020	0.225
ENSG00000102554	KLF5	FOXA1_expression	2.09E-02	2.35E-01	0.048	0.021
ENSG00000102554	KLF5	HNF4A_expression	4.72E-06	1.18E-04	0.246	0.054
ENSG00000102554	KLF5	TCF7L2_expression	2.41E-04	6.55E-03	0.269	0.073
ENSG00000102554	KLF5	MYC_expression	5.76E-02	2.01E-01	-0.093	0.049
ENSG00000102554	KLF5	KLF5_LRR	4.91E-03	7.00E-02	0.739	0.263
ENSG00000102554	KLF5	tumor%	1.88E-02	1.09E-01	0.584	0.249
ENSG00000136997	MYC	FOXA1_expression	7.19E-01	8.91E-01	0.009	0.025
ENSG00000136997	MYC	HNF4A_expression	9.23E-11	4.27E-09	0.410	0.063
ENSG00000136997	MYC	KLF5_expression	1.98E-02	8.65E-02	-0.179	0.077
ENSG00000136997	MYC	TCF7L2_expression	3.01E-03	2.53E-02	-0.262	0.088
ENSG00000136997	MYC	MYC_LRR	1.10E-04	7.00E-03	1.193	0.308
ENSG00000136997	MYC	tumor%	7.14E-02	2.89E-01	0.571	0.317
ENSG00000148737	TCF7L2	FOXA1_expression	8.79E-05	1.90E-03	0.064	0.016
ENSG00000148737	TCF7L2	HNF4A_expression	4.23E-01	6.56E-01	-0.037	0.046
ENSG00000148737	TCF7L2	KLF5_expression	2.18E-03	2.16E-02	0.158	0.052
ENSG00000148737	TCF7L2	MYC_expression	4.88E-04	6.80E-03	-0.141	0.040
ENSG00000148737	TCF7L2	TCF7L2_LRR	3.16E-07	9.42E-05	1.585	0.310
ENSG00000148737	TCF7L2	tumor%	2.54E-02	1.42E-01	0.469	0.210

Supplementary Methods

Note on samples

The study has been reviewed and approved by the Ethics Committee of the Hospital District of Helsinki and Uusimaa (HUS). Signed informed consent or authorization from the National Supervisory Authority for Welfare and Health has been obtained for all Finnish sample materials used. The use of the Danish tissue samples was approved by the Central Denmark Region Committees on Biomedical Research Ethics. Informed written consent was provided by all Danish participants. One of the 1699 studied tumor samples (c232.1T) was classified as an adenoma in a re-review by a pathologist. The 259 Danish tumors included 29 adenomas.

Array genotyping and Allelic imbalance analysis

The tumor and respective normal DNA samples were genotyped with Infinium Omni2.5-8 (Illumina Inc. and Estonian Genome Center) array. The B-Allele Frequencies and Log-R ratios were extracted with Illumina Genome Studio software and the allelic imbalance regions were calculated for all samples using BAFsegmentation[3] with default parameters. Regions with overlapping calls in both tumor and normal sample were excluded. 1% of genome with highest number of AI calls in the normal samples was excluded because of poor genotyping quality and germline copy number polymorphisms.

The AI regions gained (mean log-R ratio LRR of the markers in the region >0.06) or lost

(LRR<-0.14) were filtered to include segments with at least 2% of markers heterozygous. AI count graph was generated by counting tumors with AI on particular locus using bedtools genomecov[4] separately for gains and losses. The AI count graph was smoothed with max function in window of 10 consecutive breakpoints. Peaks were called as local maxima of the smoothed AI count graph. Peaks were ranked according to their topographic prominence, the height of the peak above the lowest contour line that surrounds it and does not contain a higher peak, in the unsmoothed AI graph. The highest peak of a chromosome have prominence equal to its height. The peaks with prominence at least 15 were reported.

The reported peaks were manually curated by searching the litterature and plausible candidate targets were annotated.

The 259 Danish tumor samples were genotyped and analysed with same methods except that normal tissues were not genotyped here but the germline heterozygous sites were obtained from prior genotyping[5].

Isochromosome calling

Tumor sample was called as having an isochromosome if at least 80% of at least one chromosome arm is called allelic imbalance and mean LRR on q-arm is at least 0.2 more than the mean LRR on p-arm.

Copy number analyses

The copy number analysis was done with Circular Binary Segmentation of Log-R-ratios after correction for genomic waves[6,7]. Region was called homozygous loss on a given tumor if the segment had at least 40 markers, mean LRR was more than 0.15 below the mean LRR of the sample and the segment did not overlap AI region with more than 25% of length. Region with at least 10 samples showing homozygous loss and no allelic imbalance in more than 5 samples were called as homozygous loss regions.

TCGA copy number data

Processed, segmented, level 3 copy number data from TCGA was downloaded from Broad firehose, analysis "analyses__2016_01_28", file "gdac.broadinstitute.org_COADREAD.Merge_snp__genome_wide_snp_6__broad_mit_edu__Level_3__segmented_scna_minus_germline_cnv_hg19__seg.Level_3.2016012800.0.0.tar.gz" Normal copy number is called for segments with -0.1 <= "Segment_mean" <= 0.1. Further details are in ftp://ftp.broadinstitute.org/pub/genepattern/modules_public_server_doc/CopyNumberInferencePipeline.pdf

Literature search for prior high resolution targets

We performed a literature search to identify which of the 38 curated peak genes have previously been identified as high-resolution (generously defined as AI peak with ten or less genes) targets of AI in CRC. First, the 38 candidate genes were checked in light of the TCGA CRC data[8], in which 9 genes (APC, ERBB2, IGF2, KLF5, MYC, PARK2, PTEN, SMAD4, and TCF7L2) were found to be high-resolution targets for AI in CRC. The remaining 30 genes were then checked from five large studies investigating AI[9–13]. Three additional genes (FGFR1, TP53, and KRAS) were reported as high-resolution AI targets in CRC. For the 30 candidate genes we also performed a PubMed search based on the title and abstract of the article to find genes with reported copy number alterations, but this search did not yield additional high-resolution candidates for AI in CRC.

Association of allelic imbalance peaks and phenotypes

Phenotype and cross-peak associations were studied with logistic regression with dependent

variable p, independent variable x indicating samples contribution to given AI peak and continuous covariate L giving the sum of the lengths of AI regions on chromosomes other than chromosome of peak x (and p, on cross-peak analysis). Variable p indicates either phenotype status or sample contribution to the other of the pair of peaks. The cross-peak analysis was done on all pairs of peaks in different chromosomes. Phenotypes are presented in Supplementary Data 8.

The model fitted is $p = \sigma(\mu + \alpha x + \beta L + \epsilon)$ using logistic link function $\sigma(t)$. The likelihood ratio test is used to test whether $\alpha = 0$and the two tailed p-values are reported. The statistical analysis was computed with plink 1.9[14].

Survival analysis was done with lifelines package[15].

Whole genome sequencing

Whole genome sequencing of 4 colon cancer cell lines (LoVo, GP5d, COLO320dm, CaCo2) 256 CRC and respective normal samples was carried out with an Illumina HiSeq 2000 and HiSeq X10 (Illumina Inc, and SciLifeLab) using a paired-end sequencing protocol. Read length was 100 bp. The median sequencing coverage at non-N reference sequence was >30×.

Paired-end reads were mapped against the 1000 Genomes Project Phase 2 reference assembly hs37d5 using Burrows-Wheeler Aligner (BWA) (version 0.6.2 or 0.7.12) with the parameters -n 0.06 and -q 5. PCR duplicates were removed using SAMtools (version 0.1.18) rmdup. Local realignment around suspected indel sites and base score quality recalibration were performed using Genome Analysis Toolkit (GATK) IndelRealigner and BaseRecalibrator (GATK version 2.3.9). These steps resulted in final sets of analysis-ready mapped reads that were used in somatic substitution, indel and structural variant calling.

For cell lines, genomic copy numbers were estimated with BIC-seq2[16] and regions were called Gain if log2.copyRatio > 0.384 and Loss if log2.copyRatio < -0.629, otherwise Level. These thresholds were chosen as local log2.copyRatio density minima closest to zero.

Somatic WGS data analysis

Somatic SNVs in each tumor were called using MuTect (version 1.1.4) with default parameters[17]. We filtered somatic SNV calls against a pooled set of whole-genome sequences from ten blood samples by excluding any somatic call which was found in three or more reads in the pooled data.

To call somatic indels, we used VarScan version 2.3.6.[18] Parameters for calling somatic variants were as follows: tumor purity 0.5, minimum variant frequency 0.05 and minimum

coverage 6. The same set of pooled blood samples were used as above. Indel call was filtered out if two or more samples had more than three reads calling an indel at 100 bp (read length) flanking the indel locus. In addition, indels outside the strict accessibility map from the 1000 Genomes Project were excluded. As a proxy for the sensitivity after filtering, we checked 80 somatic indel calls in *APC*, and noted that none of them were excluded by the above filtering steps. This observation confirms that these filtering steps do not result in a significantly increased false negative indel calling rate.

Significance analysis of the mutations within genes (Supplementary Data 9) was performed with a permutation-based method OncodriveFML[19]. Briefly, OncodriveFML attempts to identify signals of positive selection by estimating the functional impact of somatic mutations. We used OncodriveFML's default, CADD[20], as the functional impact score. Observed impact scores are then compared against expected scores generated by permuting observed mutations in a local genomic neighborhood to obtain empirical *p*-values. The default parameters of OncodriveFML were used, except that the number of permutations was increased to one million from the default of 100,000. Genes were defined with UCSC GRCh37/hg19 annotations (May 2, 2016 version), the regions also contained non-coding areas. For MSS tumor set, each chromosome was analyzed separately and the results for different chromosomes were combined before correcting for multiple testing with Benjamini-Hochberg method over all genes in each tumor set. Results are in Supplementary Data 10)

Tumor purity analysis in array data

Tumor purity was estimated with ASCAT (version 2.4.1) using default parameters and matched normal samples[21] (*104*). Before ASCAT, a separate GC-correction was performed using PennCNV[7,22,23]. First, a GC-content file was produced using UCSC hg19 GC

annotation file. The GC content file was then used to adjust the signal intensities.

The mBAF and LRR values of every AI call were plotted against the ASCAT derived tumor purity values. For gains and losses separately, an ordinary linear regression model $y=a+b*p+e$ was fitted with where y is the observed mBAF/LRR, p is the ASCAT derived tumor purity, $0\leq p\leq 1$, and the residual e was used as new variable representing mBAF/LRR values independent of tumor purity.

A weighted mean of the purity corrected LRR and mBAF values (cLRR and cBAF) was calculated for each AI peak region. The length of the overlap between an AI peak region and an AI region of an individual sample was used as a weighting factor. The analysis was performed separately for loss and gain peaks. The mean purity corrected LRR and mBAF values of the peak regions were then plotted against each other. Confidence intervals were calculated using nonparametric bootstrap with 10,000 bootstrap replicates.

Screening of KRAS hotspots by Sanger sequencing

KRAS was screened for mutations in exons 2, 3 and 4. The mutation hotspots analyzed were located in the codons 12, 13, 61, 117 and 146. Primers were designed utilizing Primer3Plus-program[24] using the reference genome GRCh37.p13 (GCA_000001405.14). PCR was performed using AmpliTaqGold DNA polymerase (Applied Biosystems, Foster City, CA) and the PCR products were purified using A'SAP PCR purification kit (ArticZymes, Tromsø, Norway). Capillary sequencing was completed in the Institute for Molecular Medicine Finland (FIMM) SeqLab (Helsinki, Finland) using BigDye v.3.1 and ABI3730xl DNA Analyzer (Applied Biosystems, Foster City, CA). The sequencing results were analyzed both manually and with Mutation Surveyor –software V4.0.8 (SoftGenetics, State College, PA).

Primers sequences:

KRAS_ex2_F
TTTGTATTAAAAGGTACTGGTGGA
KRAS_ex2_R
ATCAAAGAATGGTCCTGCAC
KRAS_ex3_F
TTTTTGAAGTAAAAGGTGCACTG
KRAS_ex3_R
GGGATATTACCTACCTCATAAACATT
KRAS_ex4_F
TGACAAAAGTTGTGGACAGGT
KRAS_ex4_R
AAGAAGCAATGCCCTCTCAA

TP53-null cell line culture

TP53 was disrupted in normal human female cell line RPE1 (from ATCC, CRL-4000) with CRISPR/Cas9. The lack of p53 protein was verified with western blot assay.

The wildtype and three single cell derived *TP53* null mutant populations (named RPE1-KO3, -KO6, -KO17) were cultured in parallel for 6 months. The cultures were split in 1:6 ratio for 46 times so the cells grew approximately $46 \cdot \log_2(6) \approx 119$ generations. The cultures went through two single cell bottlenecks (just after transfection and about one month before DNA extraction) and genomic DNA from final population was extracted. The DNA was sent to SciLifeLab Stockholm for library construction and sequencing on HiSeq 2500 instruments to >30x coverage with 126 bp Paired End.

The mRNA from was extracted using Dynabeads mRNA DIRECT kit (Life Technologies) on frozen pellets of hTERT-RPE1 cells with wild-type or knock-out p53, grown on 10 cm cell culture dishes.

RNA-sequencing libraries were prepared by NEBNext Ultra Directional RNA Library Prep Kit for Illumina (New England Biolabs) according to the instructions of the manufacturer, except using only half volumes for enzymatic reactions.

Samples were sequenced with Illumina HiSeq2000. Sequences were mapped to human genome (ENSEMBL build 75, GRCh37) using tophat2, quantified using cuffquant with rRNA and tRNA sequences masked, and analyzed by cuffdiff.

Two further clonal lines, KO6.2 and KO17.3 were derived with two weeks of culture from KO6 and KO17, and subjected to SNP array genotyping (Infinium OmniExpress, Illumina Inc.).

Cell culture, chromatin immunoprecipitation and ChIP-nexus library construction

Human LoVo (ATCC, cat. no. CCL-229), GP5d (Sigma, cat. no. 95090715) and

COLO320DM (ATCC, cat. no. CCL-220) cells were cultured in DMEM supplemented with 10% FBS and antibiotics. The chromatin immunoprecipitation (ChIP) was performed as previously described with minor modifications (*108*) and fifty millions cells were used per TF for ChIP-nexus protocol. The antibodies used for ChIP are HNF4A (sc-8987x), KLF5 (sc-22797x), FOXA1 (ab23738), MYC (Millipore, 06-340), TCF7L2 (Xalpha biologicals, X1070M), rabbit IgG (sc-2027), mouse IgG (sc-2025) and goat IgG (sc-2028).

Briefly, the cells were harvested at 80% confluence and fixed in 1% formaldehyde for 10 min at room temperature followed by addition of 0.125 M glycine. The cells were washed with ice-cold PBS twice and collected in lysis buffer (5 mM PIPES, pH 8.0, 85 mM KCl, and 0.5% NP-40). The cell suspension was centrifuged and the pellet resuspended for lysis in RIPA buffer (1% NP-40, 0.5% sodium deoxycholate, 0.1% SDS in 1 × PBS) containing protease inhibitors (Roche). The chromatin was sonicated to an average fragment size of 100–300 bp using Bioruptor (Diagenode), after which the samples were centrifuged at 13000 rpm for 15 min at +4 C to collect the supernatant. Dynal protein-G magnetic beads (Invitrogen) were pre-washed with 5mg/ml BSA in PBS wash buffer for a total of 5 times and resuspended in 1 ml of wash buffer. Antibodies specific for the protein of interest were coupled to magnetic beads overnight with rotation at +4 C. The next morning antibody-conjugated beads were washed for a total of three times in 5mg/ml BSA in PBS wash buffer and resuspended in 100 µl of wash buffer. To this, 900 µl of sonicated chromatin in RIPA buffer (from fifty million cells) were added and incubated on a rotator overnight at 4°C. After incubation, beads were washed 5 times with LiCl wash buffer (100 mM Tris- HCl, pH 7.5, 500 mM LiCl, 1% NP-40, and 1% sodium deoxycholate) and followed by two washes with 10 mM Tris-HCl (pH 7.5).

ChIP chromatin on beads were processed through ChIP-nexus protocol[25]. ChIP-nexus DNA samples with different indices were sequenced on an Illumina HiSeq4000, with single end-sequencing primer for 55 cycles.

In addition to ChIP-nexus, ChIP-exo data from human LoVo cells (ATCC, cat. no. CCL-229) using antibodies for HNF4A (sc-8987X) and KLF5 (sc-22797X) were utilized. ChIP-exo experiments were performed essentially as previously described[26] with modifications from[26,27].

Transcription factor binding site analysis

The ChIP-nexus and ChIP-exo peak calling was performed with the PeakXus-software[28] (https://github.com/hartonen/PeakXus) using the default parameter values. The ChIP-nexus reads were aligned against the hg19 reference genome using the Burrows-Wheeler alignment tool (BWA)[29] (http://bio-bwa.sourceforge.net/). BWA was evoked with the default parameters except that we set "-q 20" meaning that reads with mapping quality MAPQ<20 were discarded. The bwa aln algorithm was used for aligning. The first two bases after the nine bps long barcode (UMI plus the fixed sequence CTGA) were not aligned because of their lower average per base sequence quality compared to the rest of the read as assessed with the FastQC (http://www.bioinformatics.babraham.ac.uk/projects/fastqc/) quality control tool. UMIs were used to filter out duplicated reads during the peak calling for all ChIP-nexus experiments. Filtering was performed by counting each UMI-label only once per position. The ChIP-nexus experiments use all five base pairs long nucleotide combinations as UMI-labels, as described[25]. UMIs were used in peak calling for KLF5 ChIP-exo but not for HNF4A ChIP-exo because PeakXus only supports use of UMI-labels of fixed length. The UMI-label design of the ChIP-exo experiments is described[27]. QC metrics and enriched

motifs by MEME[30] (http://meme-suite.org/tools/meme) for each successful ChIP experiment are collected in Supplementary Tables 6-9.

PeakXus peaks with FDR <0.05 and closer than 100bp were merged across all experiments for each TF using HOMER[31] (http://homer.salk.edu/homer/). Only binding sites with the expected motif within ±100bp distance from the peak center were considered in the analyses of downstream target genes. Sequence logos of the expected motifs used in defining the binding sites are represented in Supplementary Figure 15. Topologically associated domains from human ES cell Hi-C data (H1, combined from two replicates, downloaded from http://www.3dgenome.org) were used to identify ChIP peaks nearby and potentially regulating the transcription factor targets of AI[32] (Supplementary Data 6).

siRNA silencing, and RNA-seq library preparation

The human ON-TARGETplus siRNA smartpools for silencing 38 AI targets genes along with non-targeting controls, GAPDH positive control was ordered from GE Dharmacon and resuspended in 1x siRNA buffer (GE Dharmacon). The transfection efficiency was optimised for transfection reagent and siRNA concentration for all three cell lines, LoVo, GP5d and COLO320. The cells were seeded onto 24 well plates the previous day and siRNA smartpools were transfected at 50 nM final concentration with Dharmafect 4 in biological triplicates.

The cells were harvested 48 hours after siRNA transfection for all three cell lines and total RNA isolated using RNeasy 96 kit (Qiagen) with on-column DNase digestion and eluted in RNase-free water. The isolated total RNA was quantified on Nanodrop and 0.5-1.0 μg was taken RNA-seq library preparation. The poly(A) mRNA capture and the stranded mRNA-seq library construction was done using KAPA stranded mRNA-seq kit for Illumina platforms (KAPABIOSYSTEMS) as per manufacturer's instructions. All the final individual mRNA-

seq libraries from siRNA silencing were quantified using KAPA library quantification kit for Illumina platforms (KAPABIOSYSTEMS) on Roche Lightcycler 480 and size analyzed on Fragment Analyzer (AATI) as per manufacturer's instructions for NGS libraries. Individual mRNA-seq samples with 24 different indices were pooled, quantified on Bioanalyzer (Agilent) and sequenced on an Illumina HiSeq 4000 with single-end sequencing primer for 55 cycles.

siRNA silencing RNA-seq data processing

RNA-seq data from LoVo, GP5d and COLO320 siRNA experiments was preprocessed using Kallisto (version 0.43.0) software[33]. Kallisto quantification mode was ran in strand specific mode (--rf-stranded) and aligned against the Ensembl Human reference transcriptome (ENSEMBL build 79, GRCh38, http://bio.math.berkeley.edu/kallisto/transcriptomes/). The number of bootstraps was specified as 50. Kallisto's single-end read mode (--single) requires to specify the average fragment lengths, which were estimated from Bioanalyzer (Agilent) fragment length distribution plots (avg. fragment length ranging from 353bp to 365bp). The estimated standard deviation of fragment length was set to 86 for each RNA-seq library.

Kallisto's quantification results were normalized based on the sleuth (version 0.28.1) R package[34]. Gene-level aggregation was used for the differential analysis. For LoVo, GP5d and COLO320 siRNA experiments, standard Wald tests were used to compare perturbed experiments to the control (non-targeting siRNA) experiment. In the final analysis, only experiments where the perturbation had changed the target gene with <5% FDR were taken into account.

Danish tumors, and RNA-seq data processing

The set of Danish colorectal tumor samples was described in [5]. The published subset included

the first 103 tumor-normal pairs, and the rest of the 259 sample pairs were collected and processed in the same manner.

RNA-seq data from the 259 tumors was preprocessed using Kallisto (version 0.43.0) software[33]. Kallisto quantification was ran in paired-end mode and aligned against the Ensembl Human reference transcriptome (ENSEMBL build 79, GRCh38, http://bio.math.berkeley.edu/kallisto/transcriptomes/). The number of bootstraps was specified as 25. Kallisto's quantification results were normalized based on the sleuth (version 0.28.1) R package[34]. The quantification data was processed following sleuth's default filtering settings, gene-level aggregation and response error measurement model[34]. In total 712 genes out of the 927 CRISPR/Cas9 screening genes had quantifiable RNA-seq data available after the default sleuth filtering.

The model used to test association between the estimated somatic copy-number and gene expression was `Y ~ LRR + tumor%`, where `LRR` denotes the estimated somatic copy-number (here, median log-R ratios over the AI peak region), and `tumor%` denotes the pathologists estimate tumor percentage of the tissue sample (median 0.75, mean 0.76, range 0.45-0.95). Standard Wald tests were used to compute test statistics and fold-changes for each predictor variable. Benjamini-Hochberg procedure was applied to control FDR over the 712 genes. Variance inflation factor threshold 4 was used to determine multicollinearity; none of the tests suggested multicollinearity over the two predictor variables. Results are given in Supplementary Figure 3, Supplementary Tables 2, 10 and Supplementary Data 3. In total 557/712 genes had significant association (FDR<10%) to their estimated somatic copy-number while controlling for the underlying tumor percentage. All except nine genes (*AGR2, AGR3, PDLIM3, RASL12, RBPMS2, REEP6, SLC51B, TRAF3IP3* and *ZIC2*) out of 557 had positive correlation between copy-number and expression.

The model used to test association between transcription factors' expression was `Y ~`

`predictor1_expression + predictor2_expression + ... + LRR + tumor%`, where the list of `predictor1_expression`, `predictor2_expression`, … predictor variables were given as the observed expression of *FOXA1*, *HNF4A*, *KLF5*, *MYC* and *TCF7L2* (here, log-transformed, observed counts from the default sleuth bootstrapping procedure), and `LRR` was given as the estimated somatic copy-number at the response gene. Sleuth's standard Wald tests were used to compute test statistics and fold-changes for each predictor variable (detailed results in Supplementary Table 10). Note that the model has limitations detecting double-sided regulatory interactions like, for example, *HNF4A-KLF5* interaction seen in siRNA data (Fig. 2 B), where *KLF5* had positive effect on *HNF4A* expression and *HNF4A* had inverse effect on *KLF5* expression.

CRISPRko library design

In total 24944 guide RNAs (gRNAs) were designed to target the 1928 candidate target genes in AI regions and 198 additional control genes. The coding regions of the first and second exon of all protein coding transcripts of candidate target genes annotated in Ensembl version 75 were targeted by five and three gRNAs, respectively, or up to eight editing the second if five was not found for the first. The gRNAs were chosen in the order of their on-target efficiency predicted by a knockout-efficiency model[35]. The number of other exact genomic matches of the 15b subsequence of the gRNA next to the PAM sequence was used as the count of potential off-target sites. The sgRNAs with potential off-targets (1899, 7.6%) were chosen only if the required number guides for a target exon was not found otherwise. The same rules were applied to design gRNAs for the control genes. The positive control set consisted of cell cycle regulator PLK1 and twenty strongest hits identified in a genome-wide screen of fitness genes[35,36] in HCT116 cell line that were also in the "core essential" set identified across several cell lines. The negative control set consisted of 167 randomly chosen

immunoglobulins and T-cell and odorant receptors annotated by Ensembl. The initial design also contained a set of guides targeting promoters of both protein-coding and non-coding gene that eventually were not utilized as a separate library was designed for CRISPRa experiments (see below). The oligos were designed to contain a six bases long random sequence label (RSL) before the gRNA sequence to facilitate monitoring of small subpopulations of edited cells[37] except for a subset of control guides that were added with and without the RSL label. The designed guide sequences are available in file CRC_AI_CRISPRko_oligos_2libr.Zhang_scores.tsv at Zenondo repository https://doi.org/10.5281/zenodo.1222172

CRISPRa library design

To facilitate activation of the candidate target genes, another set of 24944 gRNAs was designed. Each target gene promoter (a transcript start site annotated in Ensembl 75) was targeted by six gRNAs. The guides were designed to target the first 200 bases upstream of each TSS as previously suggested[38]. The same rules were applied as in the CRISPRko guide design except that the on-target efficiency was predicted using the CRISPRi/a model[35]. The 152 negative control genes were selected from the same set of genes as in CRISPRko design. As determining positive control genes was difficult, ten genes that gave a strong phenotype in a genome-scale CRISPRa screen[39] were added for comparison. This time the RSL was added downstream of the guide RNA so that it was not transcribed. The designed guide sequences are available in file CRC_AI_CRISPRa_1610_oligos.with_Zhang_scores.no_NONT.tsv at Zenondo repository https://doi.org/10.5281/zenodo.1222172

CRISPR screens

Library cloning

2 x 12472 sgRNA oligonucleotides (96-102nucleotides) were ordered from CustomArray and doublestranded with following PCR primers:

Ds-fw: 5’-GTATTTCGATTTCTTGGCTTTATATATCTTGTGGAAAGGACG-3’

Ds-rev 5’-CGGACTAGCCTTATTTTAACTTGC-3’

The products were then cloned into the lentiviral vector pLenti-Guide Puro (Addgene #52963) by Gibson assembly. The assembled products were transformed into electrocompetent E. coli (E. cloni Endura, Lucigen) such that about 6 million and 4,6 million colonies were obtained for Library 1 and 2, respectively. After Illumina Input library sequencing 478 and 369 x library coverage was counted for Library 1 and 2.

Library packaging

8 x 175cm2 plates of HEK293FT cells were transfected with pooled library 1 & 2 plasmids and two packaging plasmids psPAX2 (Addgene #12260) and pMD2.G (Addgene # 12259). Virus was collected after 48h and 72h and concentrated by using ultracentrifugation. Concentrated virus was stored in -80C.

Generation of Cas9 expressing CRC cell lines

GP5d, COLO320dm and CaCO2 CRC cell lines were transduced with lentivirus containing the plasmid pLenti-Cas9-Blast-sgHPRT, which contains both wildtype Cas9 and a guide sequence against the HPRT1 gene (GATGTGATGAAGGAGATGGG)[37].

48 hours after transduction, cells were selected with 4-6µg/ml Blasticidin for Cas9 expression

for 7 days, and another 14 days additionally with 5μg/ml 6-thioguanine. After selections, Cas9 expression and lack of HPRT expression was monitored by Western blotting (data not shown). 3μg/ml of Blasticidin was used for all these cells through the screens.

Library transduction & CRISPR screening

A minimum of 40 million Cas9 expressing GP5d, COLO320dm or CaCO2 cells were transduced (MOI 1) with lentiviruspool containing Libraries 1 and 2. After 48h, 1ug/ml puromycin was added to cells. First timepoint sub-culturing was performed 2 days after puromycin addition. Cells were grown for 33 days after transduction with sub-culturing being performed every 4-6 days. During sub-culturing steps, minimum of 40million cells were reseeded and also collected to freezer for the later Illumina sequencing analysis. Screens in all cell lines were performed as duplicates.

Preparation of CRISPRseq libraries from genomic DNA

Genomic DNA was isolated with Blood & Cell Culture DNA Maxi Kit (Qiagen).

200 μg of genomic DNA was used as template in 40 parallel PCR reactions (5 μg template DNA each) for 15 cycles using the primer pair 1F/1R.

After amplifications the PCR reactions were pooled and 5ul of this pool was used as template to the second PCR, which was amplified for 20 cycles with primer pair 2F/2R.

The third PCR was amplified for 15 cycles with 2ul of amplified second PCR product being used as its template. Third PCR was amplified with 3F/3R primer pair, where 3R contains the Illumina index for multiplexing, indicate by NNNNNN in the primer sequence given. All the PCR reactions were done with KAPA HiFi HotStart polymerase (KAPA Biosystems).

1F 5’-GGACTATCATATGCTTACCGTAACTTGAAAGTATTTCG-3’

1R 5’-CTTTAGTTTGTATGTCTGTTGCTATTATGTCTACTATTCTTTCC-3’

2F 5’-ACACTCTTTCCCTACACGACGCTCTTCCGATCTCTTGTGGAAAGGACGAAACAC-3’

2R 5’-AGACGTGTGCTCTTCCGATCTCTACTATTCTTTCCCCTGCACTGT-3’

3F 5’-AATGATACGGCGACCACCGAGATCTACACTCTTTCCCTACACGACGC

3R 5’-CAAGCAGAAGACGGCATACGAGATNNNNNNGTGACTGGAGTTCAGACGTGTGCTCTTCCGATCTCTAC-3’

Sequencing was then done with the custom sequencing primer CRISPR_Seq and the standard Illumina index read primer:

CRISPR_Seq 5’-CGATCTCTTGTGGAAAGGACGAAACACCG-3’

Index read primer 5’-GATCGGAAGAGCACACGTCTGAACTCCAGTCAC-3’

The resulting product of 350 bp was gel purified and sequenced on Illumina HiSeq 4000 Instrument.

CRISPRa screens

Library cloning

103 nucleotide long oligo (including 10 nucleotide long UMI-sequence) was ordered from MWG and doublestranded with following PCR primers:

UMI_gibs_ff_F: Ccaagtggcaccgagtcggt

UMI_gibs_ff_R: Gtttaaaactttatccatctttgcaggatc

Doublestranded product was then cloned by Gibson Assembly into BamHI digested lenti sgRNA(MS2)_zeo-vector (Addgene #61427). The assembled products electroporated into electrocompetent E. coli (E. cloni Endura, Lucigen) and bacterial colonies were collected after 20h incubation in +30C followed by Maxi-prep DNA purification.

2 x 12472 sgRNA oligonucleotides (84nucleotides) were ordered from CustomArray and doublestranded with following PCR primers:

Ds-fw: 5’-GTATTTCGATTTCTTGGCTTTATATATCTTGTGGAAAGGACG-3’

Ds-rev 5’-GTGATCCTCATGTTGGCCTAGCTCT-3’

The products were then pooled and cloned into the UMI-sequence containing lenti sgRNA(MS2)_zeo-vector by Gibson assembly. The assembled products were transformed into electrocompetent E. coli (E. cloni Endura, Lucigen) so that the complexity of sgRNAs in cloned library was high.

Library packaging

20 x 175cm2 plates of HEK293FT cells were transfected with pooled library plasmids and two packaging plasmids psPAX2 (Addgene #12260) and pMD2.G (Addgene # 12259). Virus was collected after 48h and 72h and concentrated by using ultracentrifugation. Concentrated virus was stored in -80C.

Generation of dCas9 expressing CRC cell lines

GP5d, COLO320dm and CaCO2 CRC cell lines were transduced (MOI 0.7 / each virus) with

lentiviruses containing the plasmids lenti MS2-P65-HSF1_Hygro (Addgene #61426) and lenti dCAS-VP64_Blast (Addgene #61425).

48 hours after transduction, cells were selected with 3-4µg/ml Blasticidin for dCas9 expression and 170-200ug/ml Hygromycin for MS2-P65-HSF1 expression for 14 days. After selections, dCas9 and MS2 expression was monitored by Western blotting (data not shown). Blasticidin and Hygromycin selections were maintained through the screens.

Library transduction & CRISPRa screening

A minimum of 40 million dCas9 expressing GP5d, COLO320dm or CaCO2 cells were transduced (MOI 1) with lentiviruspool containing pooled sgRNA library. After 48h, 100ug/ml zeocin was added to cells. First timepoint sub-culturing was performed 2 days after zeocin addition. Cells were grown for 33 days after transduction with sub-culturing being performed every 4-6 days. During sub-culturing steps, minimum of 40million cells were reseeded and also collected to freezer for the later Illumina sequencing analysis. Screens in all cell lines were performed as duplicates. 100ug/ml zeocin selection was maintained through the screens.

Preparation of CRISPRa_seq libraries from genomic DNA

Genomic DNA was isolated with Blood & Cell Culture DNA Maxi Kit (Qiagen).

200 µg of genomic DNA was used as template in 40 parallel PCR reactions (5 µg template DNA each) for 15 cycles using the primer pair 1F/1R.

After amplifications the PCR reactions were pooled and 5ul of this pool was used as template to the second PCR, which was amplified for 20 cycles with primer pair 2F/2R.

The third PCR was amplified for 15 cycles with 2ul of amplified second PCR product being used as its template. Third PCR was amplified with 3F/3R primer pair, where 3F contains the

Illumina index for multiplexing, indicate by NNNNNN in the primer sequence given. All the PCR reactions were done with KAPA HiFi HotStart polymerase (KAPA Biosystems).

1F 5’-ggactatcatatgcttaccgtaacttgaaagtatttcg-3’

1R 5’-caagacctagaaggtccattagctgcaaag-3’

2F 5’-TCTTTCCCTACACGACGCTCTTCCGATCtcttgtggaaaggacgaaacac-3’

2R 5’-AGAAGACGGCATACGAGATgtttaaaactttatccatctttgcagg -3’

3F 5’-AATGATACGGCGACCACCGAGATCTACACNNNNNNTCTTTCCCTACACGACGCTCTTCCG-3’

3R 5’-CAAGCAGAAGACGGCATACGAGATgtttaaaactttatccatc-3’

Sequencing was then done with the custom sequencing primer CRISPRa_Seq and the standard Illumina index read primer:

CRISPRa_Seq 5’-CGATCTCTTGTGGAAAGGACGAAACACCG-3’

Index read primer1 5’-gatcggaagagcacacgtctgaactccagtcac-3’

Index read primer2 5`-AATGATACGGCGACCACCGAGATCTACAC-3’

The resulting product of 354 bp was gel purified and sequenced on Illumina HiSeq 4000 Instrument.

CRISPRko screen analysis

The exact occurrences of the 16400 gRNA sequences targeting exons of 1121 protein coding genes were counted in each sequenced sample. The read counts of the gRNAs targeting promoters were omitted from the analysis. To robustly estimate the growth effects of the guides, the reads matching each gRNA sequence were divided into 16 bins based on the first two nucleotides of their RSL[37]. Those bins with less than 10 counts in total were removed

and subsequently all the guides that after this did not have all 16 bins were removed from the analysis. The growth effect of the remaining guides was estimated by the trimmed mean (20% trimming i.e. three RSLs trimmed from both ends of the distribution) of the 16 RSL bin log2 fold changes between the last and the first time point. The counts were normalized so that the average effect of all guides was zero. All gRNA sequences were mapped to the human genome (hg19) using bowtie version 1.1.2 (options -a --best -v 3 -t -f -S -p 12) and off-target scores were calculated using a previously published formula[40]. Only the guides with the off-target score >50 (14631 out of 16400 gRNAs) were selected to the gene-level analysis (and thus only genes with at least one such guide). The rank products of the guide effects in the two replicate experiments were used as inputs to gene-level analysis. The RRA (Robust Rank Aggregation, version 0.5.6.) program, part of MAGeCK package[41,42], was used to determine such genes that the gRNAs targeting them had a significant effect on growth compared to a random assignment of gRNAs to genes. RRA was run separately for positive and negative selection in each cell line with default parameter values except that the number of permutations was set to 100,000. Finally, the genes with RRA false discovery rate <10% were called selected.

Out of the 1091 analyzed genes 81 were negatively (loss suppressing growth) and 8 positively (loss increasing growth) selected, respectively, in at least one cell line during the course of the experiment. Negatively selected genes included 16 out of 21 (76%) positive control genes. Out of the 150 negative controls genes measured none were called negatively selected but two were called positively selected. Out of the 81 negatively selected genes 5 (CDK1, SARM1, PFKP, PITRM1, and RP11-192H23.4) were only called in a cell line in which the target gene region is amplified and thus the growth defect could be due to multiple cuts to the region[43]. In addition, MYC and CTCF were called both in cell lines with normal

and increased copies of these genes.

To compare the results to Cancer Dependency Map project, CRISPR Avana dataset 17Q4 was downloaded from https://figshare.com/articles/_/5520160. The mean of the copy-number corrected gene-knockout effects as inferred by CERES[44] across the 25 CRC cell lines was used to rank the effects in CRC. The CRC-specific dependencies were estimated as the difference between the mean effects in CRC and the 316 other cell lines.

CRISPRa screen analysis

The analysis of 24944 guides targeting promoters of 2093 protein-coding and non-coding genes was done as for CRISPRko screen except that the gRNAs were grouped according to the number of G and C bases in their sequence and the guide effects were normalized to zero separately within each group. This was done because we observed a dependence between average effect size and guide sequence GC-content in some of the experiments. If a group of gRNAs had less than 5 guides, the whole group was removed from the analysis. As with CRISPRko, only the gRNAs with an off-target score >50 (21222 out of 24944) were used in the gene-level analysis.

Out of the 1987 analyzed genes 6 were negatively (activation suppressing growth) and 4 positively (activation increasing growth) selected, respectively, in at least one cell line during the course of the experiment. None of the 143 negative controls genes analyzed were called selected. One of the negatively selected genes (PLK4) belonged to the group of 10 genes chosen for comparison from a previous screen.

Fragile site analyses

We analyzed the allelic imbalance data at 21 fragile sites (Supplementary Table 1) in array

data to find out fragile sites that may be differently affected by focal loss events in MSI tumors than in MSS tumors. As focal loss events, we considered losses or homozygous losses called in array data with at least one breakpoint occurring within a fragile site. To take into account the effect of site-spanning loss events, we counted how many alleles each tumor has at the fragile site that may have a focal loss event. If the site is spanned by a heterozygous loss, a maximum of one allele may be affected; if the site is spanned by a homozygous loss, there are no alleles to be affected. The number of focal loss events was then counted in each tumor/site.

Denote the number of alleles remaining after site-spanning losses at the site by n and the number of focal events observed by n_e. For each sample and site, we counted the number of alleles n_a affected by focal AI events as n_a=min(n, n_e). For instance, if there was a site-spanning heterozygous deletion (n=1) and one focal event (n_e=1), we considered the only remaining allele to be affected by the event (n_a=min(1, 1)=1). If there was no site-spanning deletion (n=2) and two focal events (n_e=2), we said that both alleles have been affected, thus assuming that the first and second event always happen in different alleles, if possible (n_a(2, 2)=2). Finally, in the case of one site-spanning event (n=1) and two observed focal events, we have na=min(1, 2)=1, or one affected allele. This analysis only considered losses - copy number gains are not considered due to the difficulty of accurately calling absolute copy numbers.

Supplementary Table 1 shows for each site the number of alleles unaffected (no ev) and affected by focal events (event) for MSS and MSI tumors. Five sites, *FHIT, CCSER1, GPHN, WWOX* and *MACROD2*, were found to be more frequently affected by focal events in MSI than MSS tumors (*p*<0.002). The opposite was observed only at *PARK2* (OR=2.68, *p*=0.003).

Supplementary References

1. Tokheim, C., Papadopoulis, N., Kinzler, K. W., Vogelstein, B. & Karchin, R. Evaluating the Evaluation of Cancer Driver Genes. (2016). doi:10.1101/060426
2. Rajaram, M. *et al.* Two Distinct Categories of Focal Deletions in Cancer Genomes. *PLoS One* **8,** e66264 (2013).
3. Staaf, J. *et al.* Segmentation-based detection of allelic imbalance and loss-of-heterozygosity in cancer cells using whole genome SNP arrays. *Genome Biol.* **9,** R136 (2008).
4. Quinlan, A. R. & Hall, I. M. BEDTools: a flexible suite of utilities for comparing genomic features. *Bioinformatics* **26,** 841–842 (2010).
5. Ongen, H. *et al.* Putative cis-regulatory drivers in colorectal cancer. *Nature* **512,** 87–90 (2014).
6. Olshen, A. B., Venkatraman, E. S., Lucito, R. & Wigler, M. Circular binary segmentation for the analysis of array-based DNA copy number data. *Biostatistics* **5,** 557–572 (2004).
7. Diskin, S. J. *et al.* Adjustment of genomic waves in signal intensities from whole-genome SNP genotyping platforms. *Nucleic Acids Res.* **36,** e126 (2008).
8. Cancer Genome Atlas Network. Comprehensive molecular characterization of human

colon and rectal cancer. *Nature* **487,** 330–337 (2012).

9. Weischenfeldt, J. *et al.* Pan-cancer analysis of somatic copy-number alterations implicates IRS4 and IGF2 in enhancer hijacking. *Nat. Genet.* **49,** 65–74 (2017).

10. Wang, H., Liang, L., Fang, J.-Y. & Xu, J. Somatic gene copy number alterations in colorectal cancer: new quest for cancer drivers and biomarkers. *Oncogene* **35,** 2011–2019 (2016).

11. Zack, T. I. *et al.* Pan-cancer patterns of somatic copy number alteration. *Nat. Genet.* **45,** 1134–1140 (2013).

12. Xie, T. *et al.* A comprehensive characterization of genome-wide copy number aberrations in colorectal cancer reveals novel oncogenes and patterns of alterations. *PLoS One* **7,** e42001 (2012).

13. Dulak, A. M. *et al.* Gastrointestinal adenocarcinomas of the esophagus, stomach, and colon exhibit distinct patterns of genome instability and oncogenesis. *Cancer Res.* **72,** 4383–4393 (2012).

14. Chang, C. C. *et al.* Second-generation PLINK: rising to the challenge of larger and richer datasets. *Gigascience* **4,** 7 (2015).

15. CamDavidsonPilon/lifelines: 0.11.1. (2017). doi:10.5281/zenodo.815943

16. Xi, R., Lee, S., Xia, Y., Kim, T.-M. & Park, P. J. Copy number analysis of whole-genome data using BIC-seq2 and its application to detection of cancer susceptibility variants. *Nucleic Acids Res.* **44,** 6274–6286 (2016).

17. Cibulskis, K. *et al.* Sensitive detection of somatic point mutations in impure and heterogeneous cancer samples. *Nat. Biotechnol.* **31,** 213–219 (2013).

18. Koboldt, D. C. *et al.* VarScan 2: somatic mutation and copy number alteration discovery in cancer by exome sequencing. *Genome Res.* **22,** 568–576 (2012).

19. Mularoni, L., Sabarinathan, R., Deu-Pons, J., Gonzalez-Perez, A. & López-Bigas, N.

OncodriveFML: a general framework to identify coding and non-coding regions with cancer driver mutations. *Genome Biol.* **17,** 128 (2016).

20. Kircher, M. *et al.* A general framework for estimating the relative pathogenicity of human genetic variants. *Nat. Genet.* **46,** 310–315 (2014).
21. Van Loo, P. *et al.* Allele-specific copy number analysis of tumors. *Proceedings of the National Academy of Sciences* **107,** 16910–16915 (2010).
22. Wang, K. *et al.* PennCNV: an integrated hidden Markov model designed for high-resolution copy number variation detection in whole-genome SNP genotyping data. *Genome Res.* **17,** 1665–1674 (2007).
23. Wang, K. *et al.* Modeling genetic inheritance of copy number variations. *Nucleic Acids Res.* **36,** e138 (2008).
24. Untergasser, A. *et al.* Primer3Plus, an enhanced web interface to Primer3. *Nucleic Acids Res.* **35,** W71–4 (2007).
25. He, Q., Johnston, J. & Zeitlinger, J. ChIP-nexus enables improved detection of in vivo transcription factor binding footprints. *Nat. Biotechnol.* **33,** 395–401 (2015).
26. Rhee, H. S. & Pugh, B. F. Comprehensive genome-wide protein-DNA interactions detected at single-nucleotide resolution. *Cell* **147,** 1408–1419 (2011).
27. Katainen, R. *et al.* CTCF/cohesin-binding sites are frequently mutated in cancer. *Nat. Genet.* **47,** 818–821 (2015).
28. Hartonen, T., Sahu, B., Dave, K., Kivioja, T. & Taipale, J. PeakXus: comprehensive transcription factor binding site discovery from ChIP-Nexus and ChIP-Exo experiments. *Bioinformatics* **32,** i629–i638 (2016).
29. Li, H. & Durbin, R. Fast and accurate short read alignment with Burrows-Wheeler transform. *Bioinformatics* **25,** 1754–1760 (2009).
30. Bailey, T. L. *et al.* MEME SUITE: tools for motif discovery and searching. *Nucleic*

Acids Res. **37,** W202–8 (2009).

31. Heinz, S. *et al.* Simple Combinations of Lineage-Determining Transcription Factors Prime cis-Regulatory Elements Required for Macrophage and B Cell Identities. *Mol. Cell* **38,** 576–589 (2010).
32. Dixon, J. R. *et al.* Topological domains in mammalian genomes identified by analysis of chromatin interactions. *Nature* **485,** 376–380 (2012).
33. Bray, N. L., Pimentel, H., Melsted, P. & Pachter, L. Near-optimal probabilistic RNA-seq quantification. *Nat. Biotechnol.* **34,** 525–527 (2016).
34. Pimentel, H. J., Bray, N., Puente, S., Melsted, P. & Pachter, L. *Differential analysis of RNA-Seq incorporating quantification uncertainty*. (2016). doi:10.1101/058164
35. Xu, H. *et al.* Sequence determinants of improved CRISPR sgRNA design. *Genome Res.* **25,** 1147–1157 (2015).
36. Hart, T. *et al.* High-Resolution CRISPR Screens Reveal Fitness Genes and Genotype-Specific Cancer Liabilities. *Cell* **163,** 1515–1526 (2015).
37. Schmierer, B. *et al.* CRISPR/Cas9 screening using unique molecular identifiers. (2017). doi:10.1101/114355
38. Konermann, S. *et al.* Genome-scale transcriptional activation by an engineered CRISPR-Cas9 complex. *Nature* **517,** 583–588 (2015).
39. Gilbert, L. A. *et al.* Genome-Scale CRISPR-Mediated Control of Gene Repression and Activation. *Cell* **159,** 647–661 (2014).
40. Sanjana, N. E., Shalem, O. & Zhang, F. Improved vectors and genome-wide libraries for CRISPR screening. *Nat. Methods* **11,** 783–784 (2014).
41. Li, W. *et al.* MAGeCK enables robust identification of essential genes from genome-scale CRISPR/Cas9 knockout screens. *Genome Biol.* **15,** 554 (2014).
42. Li, W. *et al.* Quality control, modeling, and visualization of CRISPR screens with

MAGeCK-VISPR. *Genome Biol.* **16,** 281 (2015).

43. Aguirre, A. J. *et al.* Genomic Copy Number Dictates a Gene-Independent Cell Response to CRISPR/Cas9 Targeting. *Cancer Discov.* **6,** 914–929 (2016).

44. Meyers, R. M. *et al.* Computational correction of copy number effect improves specificity of CRISPR-Cas9 essentiality screens in cancer cells. *Nat. Genet.* **49,** 1779–1784 (2017).
