# Supplementary material for: Contribution of allelic imbalance to colorectal cancer
Source: Nat Commun. 2018 Sep 10;9:3664. doi: 10.1038/s41467-018-06132-1 (PMC6131244; doi:10.1038/s41467-018-06132-1)
Supplement: Supplementary file 2 — Description of Additional Supplementary Files [file 41467_2018_6132_MOESM2_ESM.pdf]

Description of Additional Supplementary Files:

The supplementary datasets are:

1. The 165 called AI peaks annotated with overlapping genes
2. List of references justifying the curated genes
3. Expression analysis of primary tumor samples
4. Results from CRIPPR/Cas9 knock out screen
5. Results from CRISPR activation screen
6. RNAseq differential analysis results from Sleuth
7. ChIP-exo results in TADs of target Tfs
8. Phenotype data for studied samples
9. Somatic coding single nucleotide and indel variants of genes found significant in OncodriveFML analysis (FDR<10%) or associated with colorectal cancer in COSMIC called in whole genome sequences of 234 MSS CRCs. Mutations were called with MuTect (SNVs) and VarScan2 (indels), and filtered with a pool-of-normals (N=10) approach.
10. OncodriveFML gene-level analysis of somatic single nucleotide variants in whole-genome sequenced 234 microsatellite stable colorectal cancers.
